# Genomic mediators of acquired resistance to immunotherapy in metastatic melanoma

**DOI:** 10.1016/j.ccell.2025.01.009

**Published:** 2025-02-10

**Authors:** Julia Schiantarelli, Mouadh Benamar, Jihye Park, Haley E. Sax, Giacomo Oliveira, Alice Bosma-Moody, Katie M. Campbell, David Liu, Douglas B. Johnson, Scott Rodig, Catherine J. Wu, F. Stephen Hodi, Antoni Ribas, Eliezer Van Allen, Rizwan Haq

**Affiliations:** 1 Department of Medical Oncology, Dana-Farber Cancer Institute, Harvard Medical School, Boston, MA 02115, USA; 2 Broad Institute of Harvard and MIT, Cambridge, MA 02142, USA; 3 Vanderbilt University Medical Center, Nashville, TN 37232, USA; 4 Department of Pathology, Brigham and Women’s Hospital, Boston, MA 02115, USA; 5 Division of Hematology/Oncology, Department of Medicine, University of California, Los Angeles, Los Angeles, CA, USA; 6 Parker Institute for Cancer Immunotherapy, Dana-Farber Cancer Institute, Boston, MA 02215, USA; 7 These authors contributed equally; 8 These authors contributed equally; 9 Lead contact

## Abstract

Although some patients with metastatic melanoma experience durable responses to immune checkpoint inhibitors (ICIs), most exhibit intrinsic or acquired resistance to these therapies. Here, we compare somatic genomic profiles from matched pre-treatment and post-resistance tumor biopsies in patients (*n* = 25) with metastatic melanoma who exhibited heterogeneous ICI responses to nominate additional mediators of acquired resistance. We find that several acquired resistance tumors exhibit defects in *B2M* or *JAK1/2*, consistent with prior findings. We also discover resistance-associated mutations in *SEC24C* and *SEC24D* in 3 patients. SEC24 has an essential role in the trafficking of the dsDNA sensor STING and has been linked to interferonopathies. Melanoma cells engineered to express the *SEC24C* mutations observed in patients exhibit diminished STING signaling, including decreased type I interferon production, antigen presentation, and a reduced capacity to activate cytotoxic T cells. This study nominates a role for aberrant STING trafficking in acquired resistance to ICIs.

## In brief

Schiantarelli et al. compare genomic profiles from tumor biopsies of melanoma patients who exhibit heterogeneous responses to immune checkpoint inhibitors. Three patients had resistance-associated mutations in *SEC24C/D*. *SEC24* mutations exhibit diminished STING signaling, antigen presentation, and lower T cell activation. They nominate aberrant STING trafficking in acquired resistance to ICIs.

## Graphical abstract

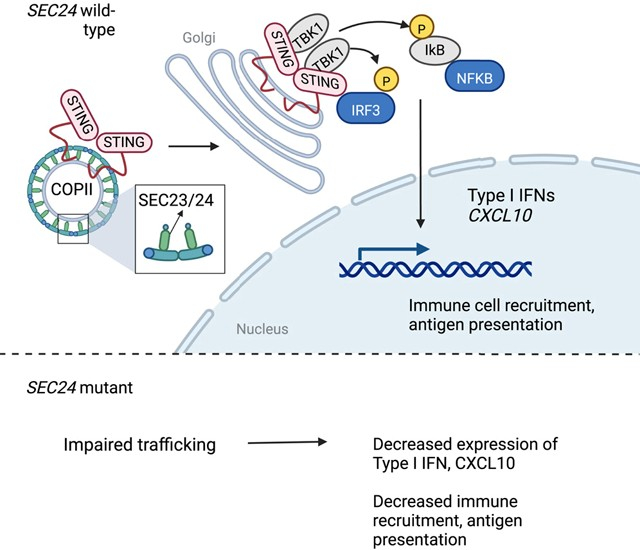

## INTRODUCTION

Immune checkpoint inhibitors (ICIs) of PD-1 and CTLA-4 have changed clinical practice for metastatic melanoma patients, but resistance to ICI occurs in most patients.^1,2^ Identifying mechanisms of resistance to ICIs has the potential to nominate approaches to restore antitumor immunity and improve the outcomes of melanoma patients. In the first published cohort of patients with acquired resistance, resistance was associated with mutations in interferon-related pathways that play a key role in upregulating antigen presentation. Specifically, mutations in the interferon receptor and *JAK1* or *JAK2* kinases were observed in 2 of 4 patients, whereas a loss-of-function (LOF) mutation in *B2M* was observed in one patient.^3^ Loss of *JAK1/2* in lung cancer was also shown to be associated with cases of acquired resistance.^4^

Building on this initial discovery, inactivation of the tumor suppressor gene *FBXW7* was also linked to resistance to anti-PD-1 therapy in one patient who exhibited heterogeneous responses,^5^ with experimental work indicating that FBXW7 regulated innate immune signaling via the dsRNA sensor RIG-I. Prior studies also identified multiple additional mechanisms of intrinsic or acquired resistance, including decreased expression of programmed death ligand 1 (PD-L1), increased upregulation of other immune checkpoints, alternative defects in antigen presentation or T cell activation, and reduced T cell trafficking and tumor infiltration or other alterations in the tumor microenvironment.^3,6–9^ Thus, antigen presentation and innate immune pathways play important roles in both intrinsic and acquired resistance to ICIs. However, the mechanisms associated with acquired resistance to ICI remain incompletely understood. Moreover, the characterization of the somatic mutations related to clinical acquired resistance to ICIs has been limited to a few patients.

Here, we compare somatic genomics of matched pre-treatment and resistant tumors from the same patient to identify candidate tumor-intrinsic resistance mechanisms. We specifically study patients who exhibited an initial clinical response to either anti-PD-1 or a combination of anti-PD-1 and anti-CTLA-4 immunotherapy, followed by recurrence (acquired resistance).

## RESULTS

**Figure 1.**
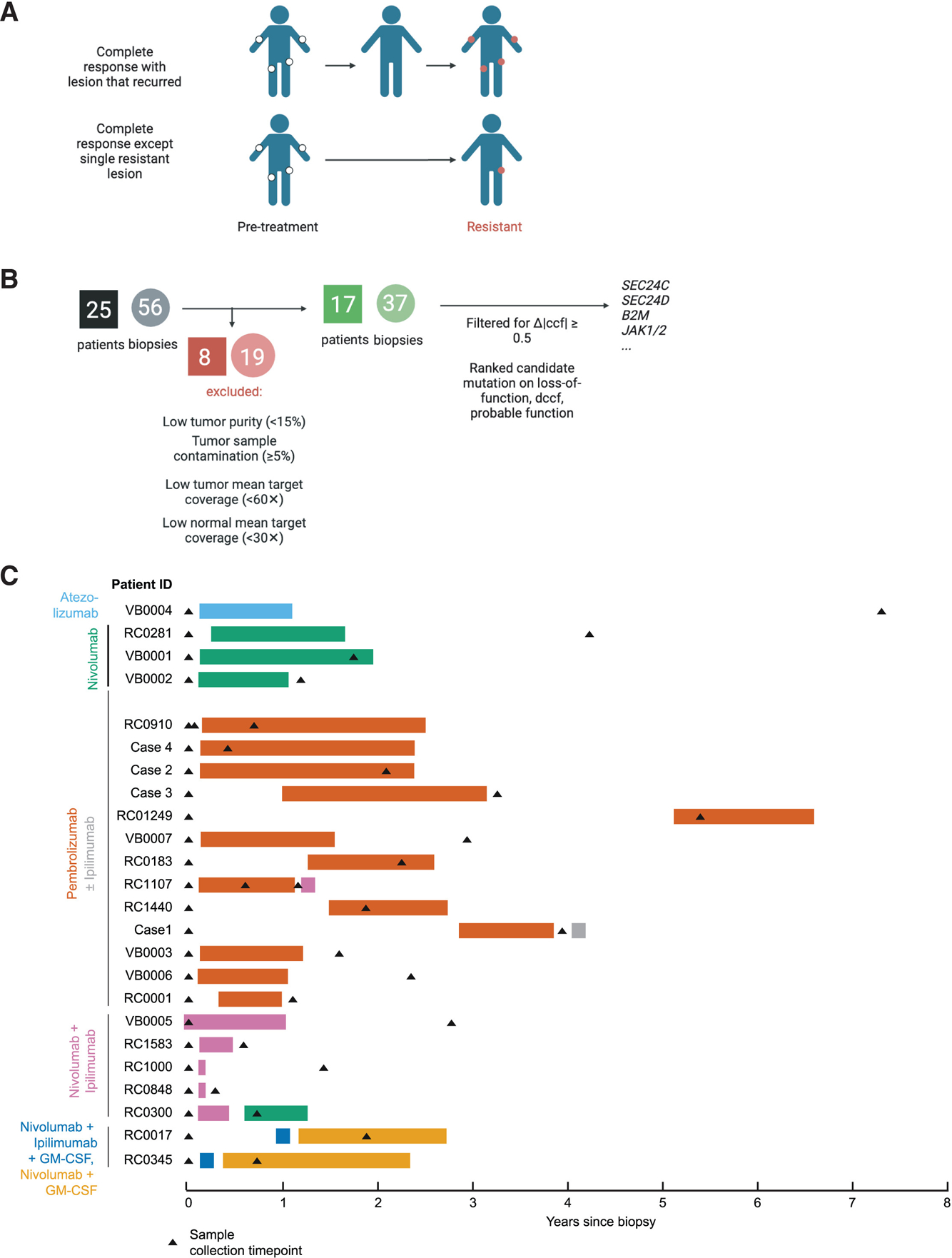
Summary of cohort selection and treatment course (A and B) An overview of the methodology of case selection (A) and subsequent analysis (B). (C) Representation of the treatment timeline for each patient and timing of paired sample collection. See also Table S1.

Cases of metastatic melanoma treated with immunotherapy were identified in which the patient had (1) a complete response to treatment except for one lesion or (2) a complete response later followed by a singular lesion that recurred (Figure 1A; Table S1). We combined our dataset with the previously published 4 cases of acquired resistance^10^ to identify occult resistance mechanisms and to more accurately measure the frequency of the previously identified mechanisms. Immunotherapies used in this cohort included pembrolizumab (*n* = 16), nivolumab (*n* = 11), atezolizumab (*n* = 1), and ipilimumab (*n* = 9) (Figure 1B; Table S1). Pre-treatment and resistant tumors, along with paired normal samples, were collected from 25 patients. In eight cases, paired samples did not pass quality control and were excluded from the primary analysis (Figure 1C; Table S1).

**Figure 2.**
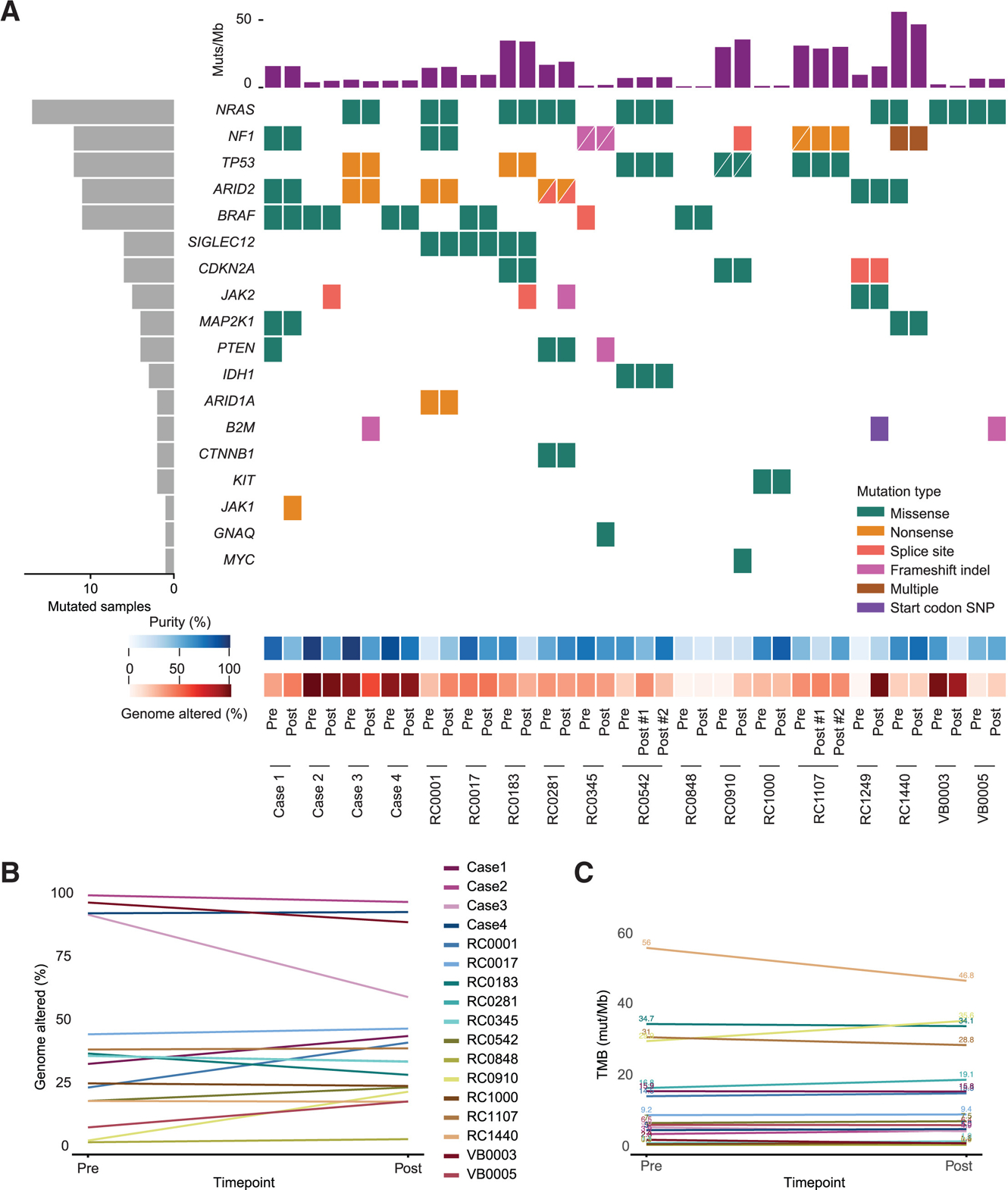
Cohort level genomic landscape (A) An overview of the genomic landscape of the cohort’s driver mutations and known drivers of acquired resistance. The CoMut plot illustrates selected single nucleotide events, tumor purity determined by ABSOLUTE, and percent genome altered. The legend indicates which kind of mutation has occurred in each gene. Each row represents the mutation status for the indicated gene, and each column represents a unique pre-treatment and resistant tumor sample from each patient. “Pre” indicates pre-treatment samples, and “Post” indicates post-treatment samples. Due to low purity, RC1249 is included only in this figure and the subsequent phylogenetic analysis for SEC24 mutations. (B) Graph of the change in percent genome altered in the pre- and post-treatment tumors, including samples with a purity > 0.15. (C) Graph showing the change in tumor mutational burden (TMB) in the pre- and post-treatment tumors, including samples with a purity > 0.15. See also Figure S1 and Table S2.

Whole-exome sequencing revealed somatic driver mutations consistent with previously described alterations,^11^ with tumors exhibiting expected alterations in *BRAF*, *NRAS*, *NF1*, *TP53*, and *PTEN* (Figure 2A). To identify genomic alterations associated with resistance, we compared sequences from pre-treatment and resistant tumors. There were no recurrent alterations found specifically in the resistant tumors (Figure 2A). Similarly, we did not observe significant differences in inferred copy-number alterations among the matched tumors (Figure 2B; Table S2). In cases with putative changes in percent genome altered, differences could be explained not by a change in copy number but rather by a lower purity of one of the two samples relative to the other. On a cohort level, no recurrent changes in copy number in the pre-treatment versus the resistant samples were noted (Figures S1A and S1B). Finally, we did not observe changes in the tumor mutational burden when comparing the pre-treatment and resistant lesions (Figure 2C; Table S2). Mutational signatures previously reported in melanoma were detected in similar abundances between matched pre-treatment and resistant samples (Figures S1C and S1D).

**Figure 3.**
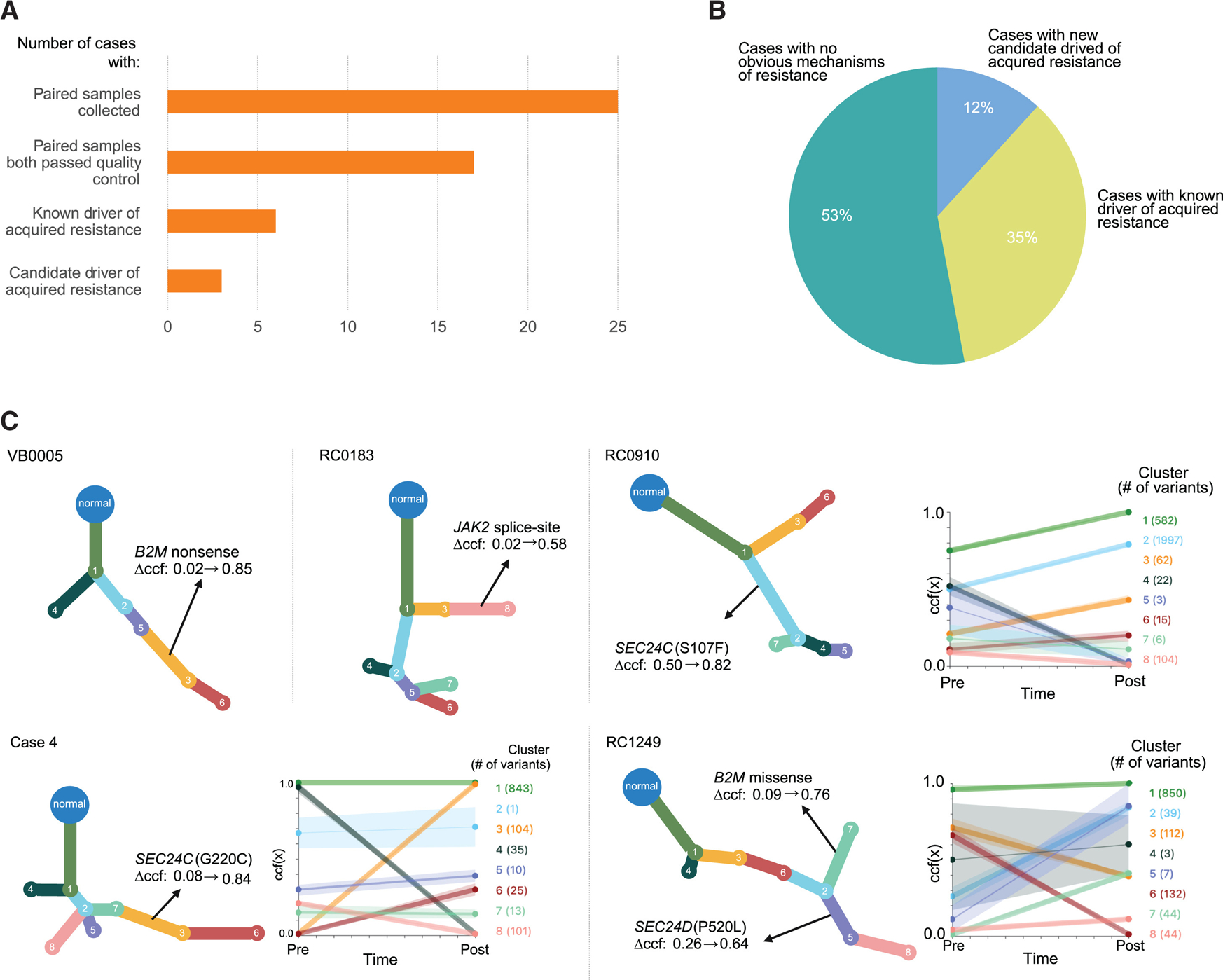
Candidate drivers of acquired resistance (A) Bar chart illustrating the number of samples collected and how many resulted in an identified candidate driver mutation. (B) Pie chart showing the fraction of cases with a known or candidate driver of acquired resistance. (C) Phylogenetic trees showing relationships between cancer cell populations in selected patients with known drivers of acquired resistance (*JAK2* and *B2M*) as well as a candidate driver of resistance (*SEC24C/D*) with corresponding graphs of the change in cancer cell fraction (CCF) of each cancer cell population including the given mutations. Each node indicates the subclone with specific mutations, and the CCF change of the same node is plotted for both pre- and post-treatment samples. See also Figures S2 and S3 and Table S3.

Among the 25 cases, including the 4 cases previously reported, only a minority of resistant tumors exhibited previously established mediators of acquired resistance after quality control (Figure 3A). Specifically, 53% (*n* = 9/17) had no previously reported driver of resistance, and 35% (*n* = 6/17) had a mutation in a gene previously implicated in acquired resistance (*B2M* or *JAK1/2*) that was expected to be deleterious and changed in cancer cell fraction (CCF) (Figure 3B). In two instances, resistance-associated mutations in *B2M* or *JAK2* were detected as subclonal events in the pre-treatment tumor and clonal in the resistant tumor (Figure 3C).

Using a biologically guided nomination procedure employed in this study to propose candidate tumor-intrinsic resistance-associated mutations (STAR Methods), we identified a mutation in *SEC24C* that increased in inferred CCF from 0.08 to 0.84 in one resistant tumor (Figures 3C and S2A). This SEC24C: p.Gly220Cys missense mutation was predicted to be loss of function using several variant discovery engines (Table S3). No other candidate resistance-associated alterations were nominated in this patient, and there were no significant changes in the percentage of genome altered or tumor mutational burden (TMB) (Figures 2B and 2C). Based on this finding, we then evaluated whether other patients without a previously reported or biologically informed mechanism of resistance had mutations in *SEC24C* or related genes. An additional case (RC0910) demonstrated a *SEC24C* missense mutation that increased in CCF from 0.50 in pre-treatment tumor samples that passed quality control to 0.82 in the resistant tumor. This SEC24C: p.Ser107Phe mutation was also predicted to be loss of function (Table S3; Figure S2B). We then extended this directed analysis to examine genes biologically related to SEC24 in paired samples with low purity (<0.2) but sufficient tumor/normal coverage (60x/30x, respectively) on each mutation site. One additional case (RC1249) harbored a *SEC24D* mutation solely in the resistant lesion. When we accounted for the purity of each sample in our phylogenetic analysis, this SEC24D:p.Pro520Leu missense mutation increased in inferred CCF from 0.26 in the pre-treatment tumor to 0.64 in the resistant tumor and was predicted to be loss of function (Table S3; Figure S2C). In this same case, we also observed a B2M:p.Met1Thr mutation in the translation initiation codon, which may affect protein translation that increased in CCF from 0.09 to 0.76 (Figure 3C). However, these mutations were phylogenetically inferred to occur in different tumor subclones. We characterized the immune microenvironment of 2 of the 3 *SEC24* mutant tumors prior to and after immunotherapy resistance (Figures S3A and S3B). We found that CD8^+^ PD1^+^ cells were diminished in the resistant samples (Figure S3C), whereas the total number of CD8^+^ cells, T cells, or other T cell subtypes did not exhibit any trends (Figures S3A–S3L). Given the complexity of immune populations and the heterogeneity of metastatic populations within a given tumor, deeper analysis of these immune cells across larger number of patients may be necessary to define immune features associated with *SEC24* mutations.

SEC24C and SEC24D are members of the COPII complex, which is necessary to export proteins from the endoplasmic reticulum (ER) to the Golgi, including stimulator of interferon genes (STING), a key mediator of the nucleic acid sensing pathway. After activation by cytosolic dsDNA, STING signaling leads to the phosphorylation of downstream kinase TANK-binding kinase 1 (TBK1) and the transcription factor interferon regulatory factor 3 (IRF3), which leads to the induction of inflammatory cytokines, including interferon β (*IFNB1*) and *CXCL10*.^12^ Consistent with these findings, individuals with defects in COP complexes have altered localization and activity of STING and immunodeficiencies and interferonopathies.^13–18^ These observations suggest that alterations in COPII, such as the SEC24C and SEC24D, may have dominant roles in immunity. Accordingly, we hypothesized that the *SEC24C* missense mutations contribute to anti-PD-1 therapy resistance through the loss of STING activity.

**Figure 4.**
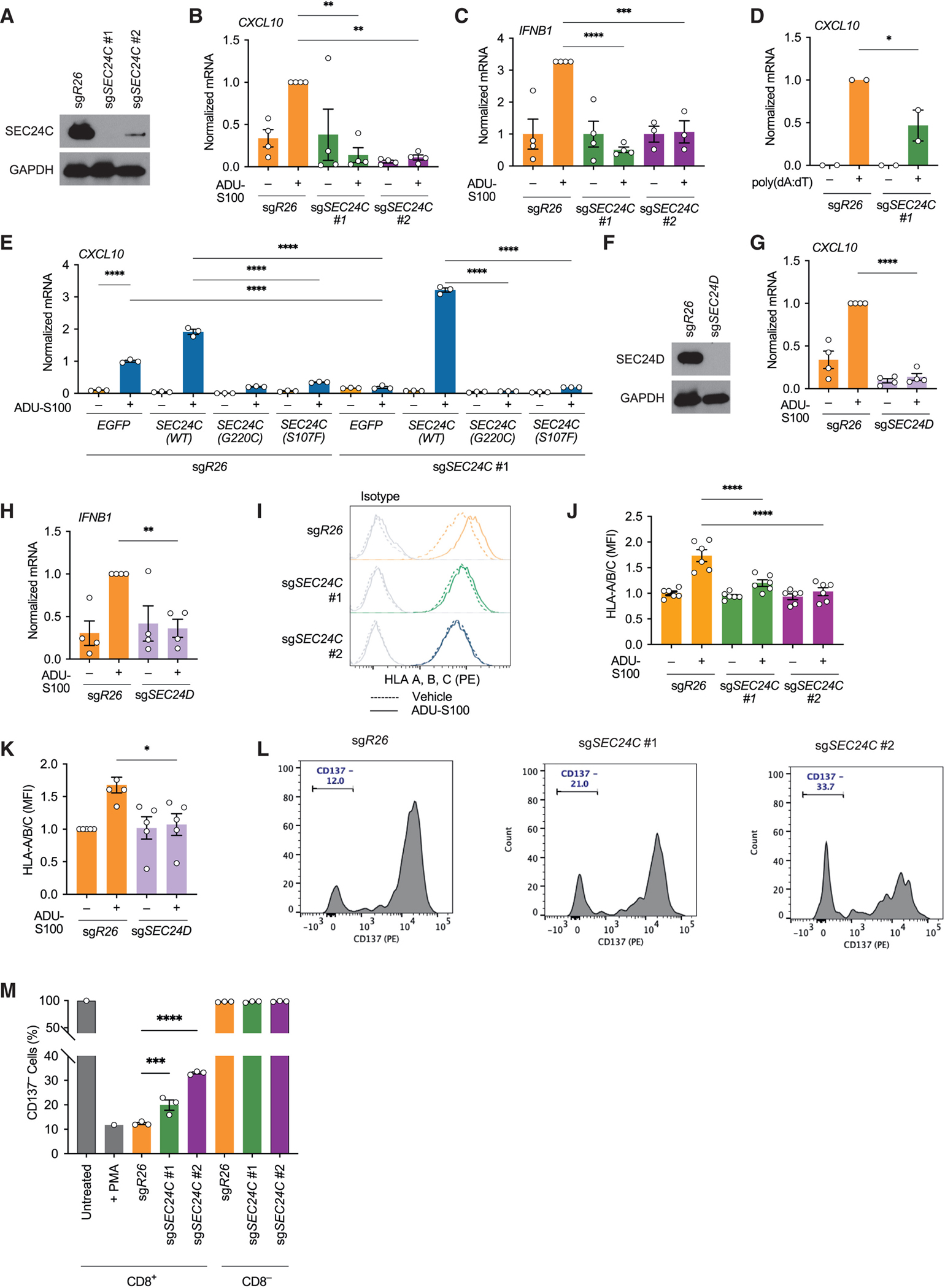
*SEC24* mutations compromise STING signaling, antigen presentation, and T cell activation (A) Western blot showing knockout of *SEC24C* in WM266.4 cells. (B and C) Relative *CXCL10* (B) and *IFNB1* (C) mRNA expression in control and *SEC24C*-deficient WM266.4 cells following treatment with STING agonist ADU-S100 (20 μM) for 4 hours. (D) Relative *CXCL10* mRNA expression in control and *SEC24C*-deficient WM266.4 cells following transfection with poly(dA:dT) (1 μg/mL). (E) Relative *CXCL10* mRNA expression in WM266.4 cells expressing *EGFP*, wild-type, or mutant *SEC24C* in control and *SEC24C*-deficient cells following treatment with ADU-S100 for 24h in a representative experiment, with individual dots representing technical replicates. (F) Western blot showing knockout of *SEC24D* in WM266.4 cells. (G and H) Relative *CXCL10* (G) and *IFNB1* (H) expression in control and *SEC24D*-deficient WM266.4 cells following treatment with STING agonist ADU-S100 (20 μM) for 4 hours. (I) Representative histogram of HLA expression in *SEC24C*-deficient WM266.4 cells. (J) Quantification of HLA expression in *SEC24C*-deficient WM266.4 cells following stimulation with ADU-S100. (K) Quantification of HLA expression in *SEC24D*-deficient WM266.4 cells following stimulation with ADU-S100. (L) Representative histogram of CD137 upregulation measured on CD8^+^ T cells after co-incubation with control and *SEC24C*-deficient WM266.4 cells. (M) Quantification of CD137^−^ in CD8^+^ or CD8^−^ (CD4^+^) T cells after incubation with control or *SEC24C*-deficient WM266.4 cells, with each dot representing technical replicates in a representative experiment. Unless otherwise noted, data are represented as mean ± SEM with each biological replicate indicated by a dot. Two-way ANOVA with Sidak multiple comparisons test was used to determine statistical significance. ****, *p* < 0.0001; ***, *p* < 0.0005; **, *p* < 0.005; *, *p* < 0.05; ns, no significance. See also Figure S4.

To define the effects of SEC24 in STING signaling in an isogenic model, we deleted *SEC24C* using CRISPR-Cas9 in a cutaneous melanoma cell line (WM266.4) (Figure 4A) and a human leukemia monocytic cell line (THP-1) (Figure S4A) and evaluated the transcriptional induction of interferon target genes following stimulation with STING agonists. In wild-type cells, two targets downstream of STING, *CXCL10* and *IFNB1*, were induced after treatment with the agonist ADU-S100. However, in the *SEC24C*-deficient WM266.4 or THP-1 cells, ADU-S100 induction of these targets was strongly impaired (Figures 4B, 4C, S4B, and S4C). Genetic depletion of *SEC24C* also strongly diminished *CXCL10* expression after transfection with poly (dA:dT), a synthetic analog of double-stranded DNA in WM266.4 or THP-1 cells (Figures 4D and S4D). These results suggest that the inactivation of *SEC24C* impairs nucleic acid sensing and interferon signaling in tumor cells. To evaluate the functional effect of the observed *SEC24C* missense mutations on STING signaling, we expressed cDNAs expressing wild-type or missense *SEC24C* mutants (G220C or S107F) in control and *SEC24C*-deficient WM266.4. We found that the expression of G220C and S107F missense mutants failed to rescue the ADU-S100-induced expression of *CXCL10* in *SEC24C*-deficient cells, whereas the expression of wild-type *SEC24C* was sufficient to rescue the phenotype (Figures 4E and S4E). Interestingly, we observed that expression of the missense mutations abolished *CXCL10* induction even in the presence of endogenous (wild-type) *SEC24C*, suggesting that these mutations may be dominant negative. To evaluate if *SEC24D* was also essential for STING-dependent effects, we similarly knocked out *SEC24D* (Figure 4F). Analogous to the effects of *SEC24C* knockout, *SEC24D*-deficient WM266.4 cells had impaired *CXCL10* and *IFNB1* induction after ADU-S100 treatment (Figures 4G and 4H).

Antigen presentation to CD8^+^ T cells is required for optimal responses to immune checkpoint inhibitors.^19^ Therefore, we evaluated the effect of *SEC24C* deletion on human leukocyte antigen (HLA) induction following ADU-S100 treatment. Unlike wild-type cells, *SEC24C*-deficient cells failed to induce HLA after STING agonist treatment (Figures 4I, 4J, and S4G). These effects could be rescued upon re-expression of wild-type but not the G220C mutant *SEC24C* (Figures S4E and S4F). Like *SEC24C*-deficient cells, *SEC24D* knockout cells had impaired HLA responses to ADU-S100 treatment (Figure 4K).

Finally, we evaluated the effects of *SEC24C* deficiency on T cell activation. We obtained MLANA-specific HLA-A2:01-restricted T cells from healthy donors and cultured them with fluorescently labeled wild-type or *SEC24C* deficient WM266.4 cells. We found that *SEC24C* knockout tumor cells had a significantly impaired ability to lead to activation of T cells, as evidenced by their expression of CD137, compared to wild-type cells (Figures 4L, 4M, and S4H–S4J). These data suggest an essential role of *SEC24* in antigen presentation and activation of T cells.

## DISCUSSION

Despite the long-standing use of ICI in melanoma, very few patients with matched pre- and post-exposure molecular profiling have been reported. In contrast, primary (intrinsic) resistance to ICI has been well characterized.^20–22^ We examined the assumption that similar mechanisms drive primary versus acquired resistance phenotypes and observed no recurrent changes in TMB or percent genome altered that could plausibly explain the resistant lesion’s growth. The mutational signatures present in each sample were also similar or unchanged. Instead, previous work on acquired resistance identified focal alterations in *JAK1/2* and *B2M* observed uniquely in the resistant tumors in three out of four cases.^10^ While we did observe additional alterations in these genes in our cohort, only 4 out of 13 new cases had alterations in these three genes that were expected to be loss of function and changed in CCF. When including the four cases previously published,^10^ 35% of patients in this cohort had alterations in these pathways at a higher CCF in the resistant sample.

Overall, most samples harbored no identifiable tumor-intrinsic driver of immune escape using the profiling strategy employed herein; thus, no highly recurrent tumor-intrinsic resistance-associated alteration was observed in this cohort. These results show that the mechanisms of intrinsic or acquired resistance to ICIs are likely more varied and complex and may be driven by the behavior of immune cells not captured by tumor-focused sequencing.^23^ In these cases, we also cannot rule out tumor-intrinsic epigenetic or transcriptional changes or alterations in the tumor microenvironment in driving resistance.

Nevertheless, we identified three cases with *SEC24C/D* mutations that may contribute to acquire resistance through the regulation of STING. The STING pathway is a major regulator of type I IFN production. The STING pathway activates an immune response when self and non-self dsDNA is detected in the cytoplasm via pattern recognition receptors (PRRs).^24^ When dsDNA is detected in the cytosol, cGAS generates cGAMP, triggering STING trafficking from the ER to the Golgi via COPII.^25^ COPII’s inner coat comprises a SAR1-SEC23-SEC24 lattice, and SEC24 specifically mediates the selective recruitment of cargo.^26^ Once STING is trafficked via COPII to the Golgi, type I IFN production is induced via IRF3 and nuclear factor-kappa B (NF-κB), which promotes antigen presentation, activates dendritic cells, and increases CD8^+^ cytotoxicity.^27,28^ STING is a master regulator of the anticancer immune response via type I IFN mediated immune infiltration, and type I IFN expression has been shown to correlate with T cell infiltration of the tumor.^29^ Studies of co-cultured tumor-immune cells have demonstrated that STING downregulation can induce resistance to effector cell-mediated killing.^30,31^ This work also suggested that decreased infiltration caused by downregulated STING was mediated by reduced expression of IFN-I target genes like *CXCL10*. STING activation can also directly lead to tumor cell death in specific cell types like malignant B cells by inducing apoptosis.^32^

Our data demonstrate that *SEC24C* is necessary for dsDNA-sensing and STING-mediated interferon signaling, consistent with prior studies suggesting the requirement of *SEC24C* in STING localization and signaling. We further showed that two SEC24C mutants, unlike the wild-type cDNA, failed to rescue STING signaling. These findings indicate that the observed G220C and S107F missense mutations are loss-of-function alterations. Although SEC24 selects cargo proteins by binding directly to ER export signals, further characterization of how these mutations lead to impaired signaling remains to be clarified.

As activation of STING signaling has been found to play a necessary role in anti-PD-1 responses, our data suggest that the mutations observed here likely contributed to resistance to anti-PD-1 therapy. Notably, we observed that other *SEC24C* and *SEC24D* alterations were observed in other cases of isolated resistance. Although some of these alterations are also predicted to act as loss-of-function mutations, functional evaluation will be necessary to assess their function. Further, clarification of how these putative heterozygous mutations, which lack evidence for loss of the wild-type allele, lead to altered STING activity, is required. Some *SEC24* mutations act dominant-negatively to inhibit trafficking.^33^ Consistent with this hypothesis, expression of the missense mutations in *SEC24C* led to decreased responses to STING activation even in the presence of wild-type (endogenous) SEC24C. Furthermore, while the observed CCF properties of *SEC24C/D* mutations in these cases suggested that these mutations may have been subclonal in the pre-treatment tumor or may be *de-novo* alterations present only in the resistant lesion, multi-regional sampling from the pre-treatment tumor would be necessary to distinguish which of these two mechanisms explain the phenotype of these cases.^34,35^ Moreover, even though we narrowed down the initial set of candidates based on the certain threshold for CCF changes (greater than 0.5; with implied shift from subclonal to clonal status) or with loss-of-function predictions, further studies in larger cohorts will refine our understanding of CCF changes in specific mutations and their functional effect on immunological resistance. Of note, while the inferred CCF of these *SEC24C* mutations is non-zero in case 4, there are zero reads for the *SEC24C* mutations in the pre-treatment samples in this case.

The loss of SEC24C/D is likely to have pleiotropic effects besides STING. However, aberrant trafficking through this pathway has been linked to pathologies associated with immune activity. For example, *COPA* syndrome, characterized by high levels of type I interferon and interferon-stimulated genes, is caused by the heterozygous mutations in *COPA*, which encodes a subunit of the COPI trafficking complex that mediates transport from the Golgi to the ER. STING localizes to the Golgi in *COPA*-deficient cells, leading to chronic IFN-I signaling that can be rescued by genetic or pharmacologic inhibition of STING. It is also interesting that DiGeorge Syndrome, characterized by the deletion of 22q11.2 (containing *SEC24C*) are characterized by immunodeficiency including a wide spectrum of T cell alterations. These prior findings further indicate that disruption of trafficking can lead to STING-dependent immune dysregulation.

The evolutionary pressure of ICI therapy may then select cells bearing mutations that confer resistance. Due to the rarity of isolated or acquired resistance, assembling a larger group of samples was challenging, making it difficult to draw meaningful conclusions on the cohort level. However, the relatively small cohort nonetheless was sufficient to identify previously discovered and putative resistance mechanisms. Future work should focus on characterizing mechanisms of acquired resistance in a cohort large enough to evaluate for recurrent patterns and to use RNA sequencing (RNA-seq) to identify changes in expression in these patients. Our work suggests that certain patients may benefit from therapies that upregulate type I IFN production to circumvent SEC24/STING-mediated resistance.

## RESOURCE AVAILABILITY

### Lead contact

Further information and requests for resources and reagents should be directed to and will be fulfilled by the lead contact, Rizwan Haq (rizwan_haq@dfci.harvard.edu).

### Materials availability

All unique reagents generated in this study are available from the lead contact unless commercially available. Plasmids generated in this study are available on Addgene.

## STAR★METHODS

### EXPERIMENTAL MODEL AND STUDY PARTICIPANT DETAILS

#### Case identification

Patients were identified by the medical record who had a complete response to immunotherapy except for one lesion or had a lesion recur after an initial complete response (Figure 1A). Paired samples from pre-treatment and resistant lesions were collected and underwent whole exome sequencing, including samples from previous study.^10^ Informed consent was obtained from all patients contributing biopsies to this study. The study was conducted in accordance with recognized ethical guidelines and approved by the Dana-Farber Cancer Institute Institutional Review Board (#05–042) or the Vanderbilt University Medical Center (#100178).

#### Cell lines

WM266.4 and THP-1 cells were cultured in RPMI and DMEM, respectively, supplemented with 10% FBS and 1% penicillin-streptomycin. Cells were incubated at 37°C in 5% CO2. Cells were tested for mycoplasma using PCR-based screening (PCR Mycoplasma Detection Kit, Cat# G238, Applied Biological Materials Inc.) biweekly. All cell lines were authenticated using the ATCC Sample Collection Kit Cell Authentication Service.

### METHOD DETAILS

#### Whole exome sequencing

Tumor-enriched tissue was dissected from FFPE tissue section slides, and concurrent DNA and RNA extraction was performed using a QIAGEN AllPrep FFPE DNA/RNA extraction kit. Library preparation and WES were performed for each sample. Peripheral lymphocytes were collected for a germline DNA sample. Hybridization and capture were performed with Illumina’s Rapid Capture Exome Kit for WES and then sequenced with Illumina HiSeq as previously described.^54^ Each run was a 76 bp paired-end, and Broad Picard Pipeline was used for de-multiplexing and data aggregation. WES from the newly sequenced cohort and previously published patients were aligned to hg19 to enable consistent downstream comparative analysis.

#### SNV and indel analysis

The paired tumor-normal samples were run through the CGA pipeline,^34^ assessing copy number alterations, indels, and SNVs while filtering out 8-OxoG sequencing artifacts, FFPE artifacts, and filtering against a panel of normals. Estimated sample contamination, sample level coverage, and the gene level depth of coverage were also calculated. Contamination was estimated using ContEST^38^ and Picard Multiple Sequence Metrics. SNVs and Indels were identified using Mutect^37^ and Strelka.^39^ Strelka indel calls were confirmed with Mutect2. FFPE and 8-library preparation false positives were eliminated and further filtered out against a panel of normals.^40^ Samples were excluded if they had low tumor purity (<15%), high tumor-in-normal contamination (≥1%), high tumor sample contamination (≥5%), low tumor mean target coverage (<60x), or low normal mean target coverage (<30x). Eight cases did not have paired samples that passed these metrics and were excluded from the downstream analyses; however, we included the RC1249 case in our CoMut plot (Figure 2A) because we used this case for the phylogenetic analysis to look for *SEC24D* CCF changes (Table S1). Selected mutations were represented in a CoMut plot.^47^

#### Copy number alteration analysis

GATK CNV^45^ and Allelic CapSeg^42^ were used to identify copy number alterations. ABSOLUTE^44^ was used to determine tumor purity, ploidy, and allelic copy number. Samples for each patient were merged to create a union set, and samples were force-called again to estimate variant allele fraction for each mutation for the paired sample mutation comparison in the phylogenetic analysis. The percent genome altered was calculated by dividing the length of segments where copy number ≠ 1 by the total length of all segments. GISTIC2.0 was used to identify recurrent copy number alterations.^48^

#### Mutational signature analysis and phylogenetic analysis

Mutational signatures were determined using deconstructSigs^49^ with COSMIC v3 signatures^55^ as the reference and a signature cutoff of 0.08. PhylogicNDT^50^ was utilized to generate estimates of each mutation’s cancer cell fraction (CCF) and the phylogenetic relationship between subclones. Each node in the phylogenetic tree indicates the subclone with specific mutations, and the CCF change of the same node is plotted for both pre- and post-treatment samples. Calculate_mutational_burden (https://github.com/brendanreardon/calculate_mutational_burden) was used to estimate the tumor mutational burden of each sample. Seventeen matched pre-treatment and resistant tumor samples passed QC, with which we could model the phylogenetic and evolutionary trajectories for these cases. Although multiple samples were collected and sequenced from case RC0910, the estimated purity for many pre-treatment samples was low, between 0 and 0.22. Thus, we chose the pre-treatment and resistant sample with the highest purity and shared clonal mutations for the phylogenetic analysis (Table S1). In addition, the pre-treatment sample from RC1249 had low purity (0.09). However, we still performed the phylogenetic analyses to check on SEC24D mutations and manually reviewed the sequencing data with Integrative Genomics Viewer (IGV: Figure S2C).

#### Identification of candidate mutations

To generate candidate mutations that may have contributed to acquired resistance, we first limited the pool of candidates to mutations with an absolute change in estimated CCF greater than 0.5 between pre-treatment and resistant samples in the same patient. In cases without an obvious driver of resistance (e.g., predicted loss of function mutations in *B2M* or *JAK/STAT* that increased in CCF), we further assessed whether the mutation was predicted to be loss of function (LOF) by its CADD (https://github.com/kircherlab/CADD-scripts) or Polyphen-2 HumDiv score (http://genetics.bwh.harvard.edu/pph2/). Then, we systematically evaluated each potential candidate for the mechanism of action and by whether the purported mechanism aligned with established roles in immunologic function. We ranked the candidates based on that mechanism if the alteration had been previously described in the cancer literature.

#### Multiplex immunofluorescence

Multiplexed immunofluorescence staining was performed with the BOND RX fully automated stainer (Leica Biosystems) as previously described.^56^ The target antigens, antibody clones, and dilutions for the two multiplex panels are listed in the key resources table. Six to seven representative images per sample were obtained using the PhenoImager HT 1.0 (Akoya Biosciences) at 20 × magnification. Following image acquisition, each field of view was spectrally unmixed and analyzed using inForm 2.6 Image Analysis Software (Akoya Biosciences). All nucleated cells within a field of view were segmented via nuclear DAPI (4′,6-diamidino-2-phenylindole) staining. Using a supervised machine-learning algorithm within the software, each cell was assigned a phenotype according to biomarker expression within its defined nuclear and membrane compartments. Positive cell identification was guided by a pathologist (SJR) and visually confirmed. Cell densities (number of positive cells per mm^2^) were calculated for each marker.

#### Generation of knockout cell lines using CRISPR-Cas9 targeting

*SEC24* knockouts were generated in WM266.4 and THP1 cell lines using CRISPR-Cas9-based gene editing. The oligonucleotides purchased from Eton Biosciences (key resources table) were annealed and cloned into lentiCRISPR v2 according to standard protocols. The plasmids generated containing *SEC24C* sgRNAs were transfected with PAX2 and pMD2.G viral packaging plasmids into HEK 293T Lenti-X cells (Takara) using TransIT-LT1. Lentivirus collected after two days was used to infect WM266.4 and THP1, followed by drug selection using puromycin (1μg/mLn). Two weeks post viral infection, the pooled puromycin-resistant population was used for validation by western blots and subsequent assays.

#### SEC24C mutagenesis and rescue

Human wild-type *SEC24C* cDNA (resistant to sg*SEC24C* #1 and sg*SEC24*C #2) in a pENTR vector was synthesized by Twist Bioscience. Site-directed mutagenesis to generate the G220C and S107F mutations was performed using In-Fusion HD Cloning Kit (Takara Bio) using oligos purchased from Eton Biosciences (key resources table). *SEC24C* cDNAs were cloned into the mammalian expression vector pMVP using the MultiSite Gateway Cloning strategy. All plasmids were verified by sequencing. Following the generation of lentivirus above, WM266.4 cells were stably infected with lentivirus, then selected with 1μg/mL of blasticidin (InVivoGen). The pooled population of blasticidin-resistant cells was used in subsequent experiments.

#### mRNA extraction and RT-qPCR

Cells were seeded in 24-well plates and treated with STING agonist ADU-S100 or transfected with synthetic double-stranded DNA analog poly(dA:dT) (1ug/mL, InVivoGen) using TransIT-LT1 (3μL per 1μg DNA). RNA was collected at the indicated times in TRIzol, and total RNA was extracted and purified using the phenol-chloroform-isopropanol extraction method. RT-qPCR was performed using the iTaq Universal SYBR Green One-Step kit, and amplification was measured with LightCycler 96. Expression levels were calculated and normalized to Actin. Primer sequences used are in the key resources table.

#### Western blot analysis

Whole-cell lysates were prepared in RIPA lysis buffer (Boston Bioproducts) supplemented with cOmplete Mini protease inhibitor and Phospho-STOP phosphatase inhibitor. BCA Protein Assay Kit was used to normalize protein quantities. Samples were denatured with SDS loading dye at 95°C for 10 min. Samples were resolved on 4–20% Criterion TGX Stain-Free Precast Gels at 100 V for 15 min, then 160 V for 45 min. Proteins were transferred to 0.2mm TransBlot Turbo Midi size nitrocellulose membrane using Trans-Blot Turbo . The membranes were blocked for one hour in 5% milk in TBST, washed for 10 min in TBS-Tween, and then incubated with primary antibodies in 5% milk in TBST at 4°C overnight. # After three 15 min washes with TBST, membranes were incubated with the secondary antibody (in 5% milk in TBST) for 1 hour at room temperature. After three washes with TBST, a chemiluminescence reaction was performed using ECL Western Blotting Substrate. Films were developed in a darkroom using a Kodak X-OMAT 2000A processor.

#### HLA detection and flow cytometry

Cells were treated with ADU-S100 (20μM) in 12-well plates. 24h or 48h later, cells were collected by trypsinization and then washed twice in FACS buffer (PBS +1% BSA). Cells are stained with 1:100 PE anti-human HLA-A/B/C antibody or IgG isotype control (PE mouse IgG2a,k) for 30 min at 4° in the dark. Samples were washed three times with FACS buffer, followed by FACS analysis. BD LSRFortessa was used for data acquisition, and the median fluorescent intensity (MFI) of PE-HLA was calculated using FlowJo using FlowJo The gating strategy is described in Figure S4.

#### T cell mediated tumor cell activation assay

A pure population of MLANA-specific effectors was generated through transduction of T cells with a MLANA-specific HLA-A*02:01-restricted TCR, as described previously.^36^ After thawing, T cells were cultured for 24h in RPMI-1640 medium containing 10% FBS, 1% Pen/Strep, IL7 (5ng/mL), and IL15 (5ng/mL). Tumor cells (WM266.4) were labeled with CellTrace Violet Stain (10uM) for 20min then washed twice in PBS and seeded in a 96-well plate at 25000 cells per well. T cells were added to the tumor cells at the indicated ratios. After 24h of incubation, media containing T cells was collected, and adherent tumors were harvested and then washed with PBS. Samples were then labeled for FACS analysis using the following antibodies for 30min at room temperature: anti-CD3/APC, anti-CD8/APC-Cy7, anti-CD137/PE, and Live/Dead Green. Samples were washed in PBS, and then data acquisition was performed using LSRFortessa and analyzed using FlowJo. T-cells were cultured without target cells as a negative control or treated with a combination of activation stimulators PMA (10ng/mL, Sigma-Aldrich) and Ionomycin (250ng/mLh) as a positive control. The gating strategy is described in Figure S4.

### QUANTIFICATION AND STATISTICAL ANALYSIS

Statistical analysis was performed using PRISM. Each data point represents a biological replicate, and data are represented as mean ± SEM, unless indicated otherwise. Statistical significance was determined via two-way ANOVA with Sidak multiple comparisons test. Significance is shown as ****, *p* < 0.0001; ***, *p* < 0.0005; **, *p* < 0.005; *, *p* < 0.05; ns, no significance.

## Supplementary Material

Paired t-test for the percent genome altered between samples from the same patient (top) and for the tumor mutational burden between samples from the same patien

Clinical and genomic information regarding patients included in this study, related to Figure 1

Loss of function prediction for each mutation, Related to Figure 3

Genomic mediators of acquired resistance to immunotherapy in metastatic melanoma

Supplemental information can be found online at https://doi.org/10.1016/j.ccell.2025.01.009.

## Figures and Tables

**KEY RESOURCES TABLE:** 

REAGENT or RESOURCE	SOURCE	IDENTIFIER

Antibodies		

anti-SEC24C	Thermo Fisher Scientific	Cat# PA5-59101; RRID: AB_2647077
anti-SEC24D	Bethyl	Cat# A304-813A-T; RRID: AB_2621008
anti-GAPDH	Cell Signaling Technology	Cat# 3683; RRID: AB_1642205
HLA-A/B/C antibody	BioLegend	Cat# 311406; RRID: AB_314875
PE mouse IgG2a,k	BD Bioscience	Cat# 554648; RRID: AB_395491
anti- CD3/APC	BioLegend	Cat# 317318; RRID: AB_1937212
anti-CD8/APC-Cy7	BioLegend	Cat# 344714; RRID: AB_2044006
anti- CD137/PE	BioLegend	Cat# 309803; RRID: AB_314782
Anti-CD8 (Clone: 4B11)	Leica	Cat# NCL-CD8-4B11; RRID: AB_442068
Anti-PD-L1 (Clone: E1L3N)	Cell Signaling Technology	Cat# 13684; RRID: AB_2687655
Anti-FOXP3 (Clone: D2W8E)	Cell Signaling Technology	Cat# 98377; RRID: AB_2747370
Anti-PD-1 (Clone: EPR4877(2)	Abcam	Cat# ab137132; RRID: AB_2894867
Anti-CD11c (Clone: D3V1E)	Cell Signaling Technology	Cat# 45581; RRID: AB_2799286
Anti-CD4 (Clone: EPR6855)	Abcam	Cat# ab133616; RRID: AB_2750883
Anti-CD20 (Clone: L26)	Dako	Cat# GA60461-2
Anti-CD3	Dako	Cat# GA50361-2
Anti-CD68 (Clone: D4B9C)	Cell Signaling Technology	Cat# 97778; RRID: AB_2928056
Anti-SOX10 (Clone: EP268)	Epitomics	Cat# AC-0237; RRID: AB_2941085

Bacterial and virus strains		

One Shot^™^ Stbl3^™^ Chemically Competent *E. coli*	Invitrogen	Cat# C737303

Biological samples		

Patient tumor samples	Dana Farber Cancer Institute	See Table S1
Patient tumor samples	Vanderbilt University Medical Center	See Table S1

Chemicals, peptides, and recombinant proteins		

ADU-S100 STING agonist	Chemietek	Cat# CT-ADUS100
Poly(dA:dT) naked (synthetic double-stranded DNA)	InVivoGen	Cat# tlrl-patn
CellTrace Violet Stain	Invitrogen	Cat# C34557A
Live/Dead Green Fixable Green Dead Cell Stain	Invitrogen	Cat# L34970A
PMA	Sigma-Aldrich	Cat# P1585
Ionomycin	Sigma-Aldrich	Cat# 200-664-3
Interleukin-7 human	Sigma-Aldrich	Cat# I5896
Interleukin-15 human	Sigma-Aldrich	Cat# I8648
TRIzol	Invitrogen	Cat# 15596018
Pure Ethanol 190 Proof	Decon Labs Inc.	Cat# UN1170
Isopropanol	Fisher Bioreagents	Cat# BP2618-1
Chloroform	ChemCruz	Cat# sc-239527A
2-Mercaptoethanol	Sigma	Cat# M6250
4x Laemmli Sample Buffer	BioRad	Cat# 1610747
Fetal Bovine Serum	Gemini Bio	Cat#100-106-500
penicillin-streptomycin antibiotics	Life Technologies	Cat#15140122
Puromycin selection antibiotic	InVivoGen	Cat# ant-pr-1
Blasticidin selection antibiotic	InVivoGen	Cat# ant-bl-1
Bovine Serum Albumin	Boston BioProducts	Cat# C-11070D
Polybrene	Santa Cruz	Cat# SC-134220

Critical commercial assays		

PCR Mycoplasma Detection Kit	Applied Biological Materials Inc.	Cat# G238
iTaq Universal SYBR Green One-Step kit	Bio-Rad	Cat# 1725150

Deposited data		

Raw sequence data	This paper	dbGaP phs000452.v5

Experimental models: Cell lines		

Human: WM266.4	Cory Johannessen from Broad Institute of Harvard and MIT	N/A
Human: THP-1	ATCC	Cat# TIB-202
Human: HEK 293T Lenti-X cells	Takara	Cat# 632180
MLANA-specific HLA- A*02:01-restricted TCR	Oliveira, G. et al.^36^	N/A

Oligonucleotides		

sgRNA: targeting human *ROSA26:* ACAGCAAGTTGTCTAACCCG	Eton Biosciences	N/A
sgRNA #1: targeting human *SEC24C:* AAGAGCCCCACCTTCCTCGG	Eton Biosciences	N/A
sgRNA #2: targeting human *SEC24C* CTGTGGGCAACCAGCCACCT	Eton Biosciences	N/A
sgRNA: targeting human *SEC24D:* TTTATGCAGTTCATCGAAGG	Eton Biosciences	N/A
qPCR Primer: human *SEC24C* Forward (5’–3’): TACGGGCACAGAGATCCCGG Reverse (5’–3’): GCTTACCGGATCCGCCACCA	Eton Biosciences	N/A
qPCR Primer: human *ACTB* Forward (5’–3’): GTTGTCGACGACGAGCG Reverse (5’–3’): GCACAGAGCCTCGCCTT	Eton Biosciences	N/A
qPCR Primer: human *IFNB1* Forward (5’–3’): TTGTTGAGAACCTCCTGGCT Reverse (5’–3’): TGACTATGGTCCAGGCACAG	Eton Biosciences	N/A
qPCR Primer: human *CXCL10* Forward (5’–3’): CCACGTGTTGAGATCATTGCT Reverse (5’–3’): TGCATCGATTTTGCTCCCCT	Eton Biosciences	N/A
Mutagenesis Primer: human *SEC24C G220C:* Forward: TTCAGGGTGTCCTCGGCTGCC Reverse: CCGAGGACACCCTGAAGCTG	Eton Biosciences	N/A
Mutagenesis Primer: human *SEC24C S107F:* Forward: GGGTTTCAGCCATTTGGGTCC Reverse: AAATGGCTGGGACCCAGGCA	Eton Biosciences	N/A

Recombinant DNA		

Plasmid: lentiCRISPR v2	Addgene	Cat# 52961
Plasmid: pENTR-SEC24C	Twist Bioscience	N/A
Plasmid: PAX2 viral packaging vector	Addgene	Cat#12260
Plasmid: pMD2.G viral packaging plasmid	Addgene	Cat#12259

Software and algorithms		

FlowJo	FlowJo LLC	v10.8.1
PRISM	GraphPad	v10
The Getz Lab CGA WES Characterization pipeline employs the following tools: MuTect,^37^ ContEst,^38^ Strelka,^39^ Orientation Bias Filter,^40^ DeTiN,^41^ AllelicCapSeg,^42^ MAFPoNFilter,^43^ ABSOLUTE,^44^ GATK,^45^ and Oncotator.^46^	Broad Institute	https://portal.firecloud.org/tmethods/getzlab/CGA_WES_Characterization_Pipeline_v0.1_Dec2018/
CoMut	Crowdis et al.^47^	https://github.com/vanallenlab/comut
GISTIC2.0	Mermal et al.^48^	https://github.com/broadinstitute/gistic2
deconstructSigs	Rosenthal et al.^49^	https://github.com/raerose01/deconstructSigs
PhylogicNDT	Leshchiner et al.^50^	https://github.com/broadinstitute/PhylogicNDT
Calculate mutational burden		https://github.com/brendanreardon/calculate_mutational_burden
Integrative Genomics Viewer	Robinson et al.^51^	https://github.com/igvteam/igv
CADD	Kircher et al.^52^	https://github.com/kircherlab/CADD-scripts
Polyphen-2 HumDiv	Adzhubei et al.^53^	http://genetics.bwh.harvard.edu/pph2

Other		

TransIT-LTI Transfection Reagent	Mirus Bio LLC	Cat# MIR 2300
In-Fusion HD Cloning Kit	Takara Bio	Cat# 639650
MultiSite Gateway Cloning kit	ThermoFisher	Cat#12538120
iTaq Universal SYBR Green One-Step kit	Bio-Rad	Cat# 1725151
RIPA lysis buffer	Boston Bioproducts	Cat# BP-115
cOmplete Mini protease inhibitor	Roche	Cat# 11836153001
Phospho-STOP phosphatase inhibitor	Roche	Cat# 4906845001
BCA Protein Assay Kit	Thermo Scientific	Cat# 23224/23228
4–20% Criterion TGX Stain- Free Precast Gels	BioRad	Cat# 5678095
Trans- Blot Turbo	BioRad	Cat# 1704271
Tris Buffered Saline Tween	Boston Bioproducts	Cat# IBB-180X
ECL Western Blotting Substrate	Pierce	Cat# 32106
CellTrace Violet Stain	Invitrogen	Cat# C34557A
Live/Dead Green	Invitrogen	Cat# L34970A
Tris Glycine-SDS Running Buffer	Boston BioProducts	Cat# BP-150
Plasmid Plus Mini Kit	QIAGEN	Cat# 27106
Plasmid Plus Midi Kit	QIAGEN	Cat# 12945
DMEM, high glucose	Gibco	Cat# 11965092
RPMI 1640 Medium	Gibco	Cat# 11875093
Trypsin-EDTA (0.05%), phenol red	Gibco	Cat# 25300054
LB Broth	Lennox	Cat# P-830
LB Agar	Lennox	Cat# P-820
Dry Milk Powder	Research Products International	Cat# M17200-1000.0
Opti-MEM^™^ I Reduced Serum Medium	Gibco	Cat# 31985070
PBS, pH 7.4	Gibco	Cat# 10010023
12 Well Tissue Culture Plate, Sterile	CellTreat Scientific Products	Cat# 229111
6 Well Tissue Culture Plate, Sterile	CellTreat Scientific Products	Cat# 229105
96 Well Tissue Culture Plate, Sterile – Flat bottom	CellTreat Scientific Products	Cat# 229195
96 Well Tissue Culture Plate, Sterile – Round bottom	CellTreat Scientific Products	Cat# 229590
24 Well Tissue Culture Plate	Corning Incorporated	Cat# 3526

## Data Availability

All the data analysis information is available in our method section. Raw sequence data generated in this study are available in dbGaP (accession phs000452.v5).

## Highlights

- Somatic mutations leading to immunotherapy resistance are poorly characterized
- Resistance-associated genomic mutations in *SEC24C/D* were observed in patients
- *SEC24* mutations exhibited diminished STING signaling and innate immune signaling
- SEC24-mutant cells exhibited lower antigen presentation and activation of T cells

## References

[1] Leach DR, Krummel MF, and Allison JP (1996). Enhancement of antitumor immunity by CTLA-4 blockade. Science 271, 1734–1736. 10.1126/science.271.5256.1734. 8596936

[2] Tawbi HA, Forsyth PA, Algazi A, Hamid O, Hodi FS, Moschos SJ, Khushalani NI, Lewis K, Lao CD, Postow MA, (2018). Combined Nivolumab and Ipilimumab in Melanoma Metastatic to the Brain. N. Engl. J. Med. 379, 722–730. 10.1056/NEJMoa1805453. 30134131 PMC8011001

[3] Sade-Feldman M, Jiao YJ, Chen JH, Rooney MS, Barzily-Rokni M, Eliane JP, Bjorgaard SL, Hammond MR, Vitzthum H, Blackmon SM, (2017). Resistance to checkpoint blockade therapy through inactivation of antigen presentation. Nat. Commun. 8, 1136. 10.1038/s41467-017-01062-w. 29070816 PMC5656607

[4] Gettinger S, Choi J, Hastings K, Truini A, Datar I, Sowell R, Wurtz A, Dong W, Cai G, Melnick MA, (2017). Impaired HLA Class I antigen processing and presentation as a mechanism of acquired resistance to immune checkpoint inhibitors in lung cancer. Cancer Discov. 7, 1420–1435. 10.1158/2159-8290.CD-17-0593. 29025772 PMC5718941

[5] Gstalder C, Liu D, Miao D, Lutterbach B, DeVine AL, Lin C, Shettigar M, Pancholi P, Buchbinder EI, Carter SL, (2020). Inactivation of Fbxw7 impairs dsRNA sensing and confers resistance to PD-1 blockade. Cancer Discov. 10, 1296–1311. 10.1158/2159-8290.CD-19-1416. 32371478 PMC8802534

[6] Liu D, Schilling B, Liu D, Sucker A, Livingstone E, Jerby-Arnon L, Zimmer L, Gutzmer R, Satzger I, Loquai C, (2019). Integrative molecular and clinical modeling of clinical outcomes to PD1 blockade in patients with metastatic melanoma. Nat. Med. 25, 1916–1927. 10.1038/s41591-019-0654-5. 31792460 PMC6898788

[7] Spranger S, Bao R, and Gajewski TF (2015). Melanoma-intrinsic β-catenin signalling prevents anti-tumour immunity. Nature 523, 231–235. 10.1038/nature14404. 25970248

[8] Peng W, Chen JQ, Liu C, Malu S, Creasy C, Tetzlaff MT, Xu C, McKenzie JA, Zhang C, Liang X, (2016). Loss of PTEN Promotes Resistance to T Cell-Mediated Immunotherapy. Cancer Discov. 6, 202–216. 10.1158/2159-8290.CD-15-0283. 26645196 PMC4744499

[9] Mariathasan S, Turley SJ, Nickles D, Castiglioni A, Yuen K, Wang Y, Kadel EE III, Koeppen H, Astarita JL, Cubas R, (2018). TGFb attenuates tumour response to PD-L1 blockade by contributing to exclusion of T cells. Nature 554, 544–548. 10.1038/nature25501. 29443960 PMC6028240

[10] Zaretsky JM, Garcia-Diaz A, Shin DS, Escuin-Ordinas H, Hugo W, Hu-Lieskovan S, Torrejon DY, Abril-Rodriguez G, Sandoval S, Barthly L, (2016). Mutations Associated with Acquired Resistance to PD-1 Blockade in Melanoma. N. Engl. J. Med. 375, 819–829. 10.1056/NEJMoa1604958. 27433843 PMC5007206

[11] Conway JR, Dietlein F, Taylor-Weiner A, AlDubayan S, Vokes N, Keenan T, Reardon B, He MX, Margolis CA, Weirather JL, (2020). Integrated molecular drivers coordinate biological and clinical states in melanoma. Nat. Genet. 52, 1373–1383. 10.1038/s41588-020-00739-1. 33230298 PMC8054830

[12] Zhang L, Wei X, Wang Z, Liu P, Hou Y, Xu Y, Su H, Koci MD, Yin H, and Zhang C (2023). NF-κB activation enhances STING signaling by altering microtubule-mediated STING trafficking. Cell Rep. 42, 112185. 10.1016/j.celrep.2023.112185. 36857187

[13] Rivara S, and Ablasser A (2020). COPA silences STING. J. Exp. Med. 217, e20201517. 10.1084/jem.20201517. 32991673 PMC7527967

[14] Deng Z, Chong Z, Law CS, Mukai K, Ho FO, Martinu T, Backes BJ, Eckalbar WL, Taguchi T, and Shum AK (2020). A defect in COPI-mediated transport of STING causes immune dysregulation in COPA syndrome. J. Exp. Med. 217, e20201045. 10.1084/jem.20201045. 32725126 PMC7596814

[15] Lepelley A, Martin-Niclós MJ, Le Bihan M, Marsh JA, Uggenti C, Rice GI, Bondet V, Duffy D, Hertzog J, Rehwinkel J, (2020). Mutations in COPA lead to abnormal trafficking of STING to the Golgi and interferon signaling. J. Exp. Med. 217, e20200600. 10.1084/jem.20200600. 32725128 PMC7596811

[16] Mukai K, Ogawa E, Uematsu R, Kuchitsu Y, Kiku F, Uemura T, Waguri S, Suzuki T, Dohmae N, Arai H, (2021). Homeostatic regulation of STING by retrograde membrane traffic to the ER. Nat. Commun. 12, 61. 10.1038/s41467-020-20234-9. 33397928 PMC7782846

[17] Taguchi T, Mukai K, Takaya E, and Shindo R (2021). STING Operation at the ER/Golgi Interface. Front. Immunol. 12, 646304. 10.3389/fimmu.2021.646304. 34012437 PMC8126659

[18] Steiner A, Hrovat-Schaale K, Prigione I, Yu CH, Laohamonthonkul P, Harapas CR, Low RRJ, De Nardo D, Dagley LF, Mlodzianoski MJ, (2022). Deficiency in coatomer complex I causes aberrant activation of STING signalling. Nat. Commun. 13, 2321. 10.1038/s41467-022-29946-6. 35484149 PMC9051092

[19] Sharma P, Goswami S, Raychaudhuri D, Siddiqui BA, Singh P, Nagarajan A, Liu J, Subudhi SK, Poon C, Gant KL, (2023). Immune checkpoint therapy-current perspectives and future directions. Cell 186, 1652–1669. 10.1016/j.cell.2023.03.006. 37059068

[20] Gide TN, Wilmott JS, Scolyer RA, and Long GV (2018). Primary and Acquired Resistance to Immune Checkpoint Inhibitors in Metastatic Melanoma. Clin. Cancer Res. 24, 1260–1270. 10.1158/1078-0432.CCR-17-2267. 29127120

[21] Schoenfeld AJ, and Hellmann MD (2020). Acquired Resistance to Immune Checkpoint Inhibitors. Cancer Cell 37, 443–455. 10.1016/j.ccell.2020.03.017. 32289269 PMC7182070

[22] Kalbasi A, and Ribas A (2020). Tumour-intrinsic resistance to immune checkpoint blockade. Nat. Rev. Immunol. 20, 25–39. 10.1038/s41577-019-0218-4. 31570880 PMC8499690

[23] Keenan TE, Burke KP, and Van Allen EM (2019). Genomic correlates of response to immune checkpoint blockade. Nat. Med. 25, 389–402. 10.1038/s41591-019-0382-x. 30842677 PMC6599710

[24] Ma Z, and Damania B (2016). The cGAS-STING defense pathway and its counteraction by viruses. Cell Host Microbe 19, 150–158. 10.1016/j.chom.2016.01.010. 26867174 PMC4755325

[25] Decout A, Katz JD, Venkatraman S, and Ablasser A (2021). The cGAS-STING pathway as a therapeutic target in inflammatory diseases. Nat. Rev. Immunol. 21, 548–569. 10.1038/s41577-021-00524-z. 33833439 PMC8029610

[26] Stagg SM, LaPointe P, Razvi A, Gürkan C, Potter CS, Carragher B, and Balch WE (2008). Structural basis for cargo regulation of COPII coat assembly. Cell 134, 474–484. 10.1016/j.cell.2008.06.024. 18692470 PMC2649882

[27] Chen C, and Xu P (2023). Cellular functions of cGAS-STING signaling. Trends Cell Biol. 33, 630–648. 10.1016/j.tcb.2022.11.001. 36437149

[28] McNab F, Mayer-Barber K, Sher A, Wack A, and O’Garra A (2015). Type I interferons in infectious disease. Nat. Rev. Immunol. 15, 87–103. 10.1038/nri3787. 25614319 PMC7162685

[29] Corrales L, Matson V, Flood B, Spranger S, and Gajewski TF (2017). Innate immune signaling and regulation in cancer immunotherapy. Cell Res. 27, 96–108. 10.1038/cr.2016.149. 27981969 PMC5223230

[30] Tan YS, Sansanaphongpricha K, Xie Y, Donnelly CR, Luo X, Heath BR, Zhao X, Bellile E, Hu H, Chen H, (2018). Mitigating SOX2-potentiated immune escape of Head and Neck Squamous Cell Carcinoma with a STING-inducing nanosatellite vaccine. Clin. Cancer Res. 24, 4242–4255. 10.1158/1078-0432.CCR-17-2807. 29769207 PMC6125216

[31] Vanpouille-Box C, Demaria S, Formenti SC, and Galluzzi L (2018). Cytosolic DNA sensing in organismal tumor control. Cancer Cell 34, 361–378. 10.1016/j.ccell.2018.05.013. 30216189

[32] Tang C-HA, Zundell JA, Ranatunga S, Lin C, Nefedova Y, Del Valle JR, and Hu CCA (2016). Agonist-mediated activation of STING induces apoptosis in malignant B cells. Cancer Res. 76, 2137–2152. 10.1016/j.ccell.2018.05.013. 26951929 PMC4873432

[33] Farhan H, Reiterer V, Korkhov VM, Schmid JA, Freissmuth M, and Sitte HH (2007). Concentrative export from the endoplasmic reticulum of the gamma-aminobutyric acid transporter 1 requires binding to SEC24D. J. Biol. Chem. 282, 7679–7689. 10.1074/jbc.M609720200. 17210573

[34] Schiantarelli J, Pappa T, Conway J, Crowdis J, Reardon B, Dietlein F, Huang J, Stanizzi D, Carey E, Bosma-Moody A, (2022). Mutational Footprint of Platinum Chemotherapy in a Secondary Thyroid Cancer. JCO Precis. Oncol. 6, e2200183. 10.1200/PO.22.00183. 36075011 PMC9489159

[35] Grzywa TM, Paskal W, and W1odarski PK. (2017). Intratumor and intertumor heterogeneity in melanoma. Transl. Oncol. 10, 956–975. 10.1016/j.tranon.2017.09.007. 29078205 PMC5671412

[36] Oliveira G, Stromhaug K, Klaeger S, Kula T, Frederick DT, Le PM, Forman J, Huang T, Li S, Zhang W, (2021). Phenotype, specificity and avidity of antitumour CD8+ T cells in melanoma. Nature 596, 119–125. 10.1016/j.ccell.2023.08.013. 34290406 PMC9187974

[37] Cibulskis K, Lawrence MS, Carter SL, Sivachenko A, Jaffe D, Sougnez C, Gabriel S, Meyerson M, Lander ES, and Getz G (2013). Sensitive detection of somatic point mutations in impure and heterogeneous cancer samples. Nat. Biotechnol. 31, 213–219. 10.1038/nbt.2514. 23396013 PMC3833702

[38] Cibulskis K, McKenna A, Fennell T, Banks E, DePristo M, and Getz G (2011). ContEst: estimating cross-contamination of human samples in next-generation sequencing data. Bioinformatics 27, 2601–2602. 10.1093/bioinformatics/btr446. 21803805 PMC3167057

[39] Saunders CT, Wong WSW, Swamy S, Becq J, Murray LJ, and Cheetham RK (2012). Strelka: accurate somatic small-variant calling from sequenced tumor-normal sample pairs. Bioinformatics 28, 1811–1817. 10.1093/bioinformatics/bts271. 22581179

[40] Costello M, Pugh TJ, Fennell TJ, Stewart C, Lichtenstein L, Meldrim JC, Fostel JL, Friedrich DC, Perrin D, Dionne D, (2013). Discovery and characterization of artifactual mutations in deep coverage targeted capture sequencing data due to oxidative DNA damage during sample preparation. Nucleic Acids Res. 41, e67. 10.1093/nar/gks1443. 23303777 PMC3616734

[41] Taylor-Weiner A, Stewart C, Giordano T, Miller M, Rosenberg M, Macbeth A, Lennon N, Rheinbay E, Landau DA, Wu CJ, and Getz G (2018). DeTiN: overcoming tumor-in-normal contamination. Nat. Methods 15, 531–534. 10.1038/s41592-018-0036-9. 29941871 PMC6528031

[42] Landau DA, Carter SL, Stojanov P, McKenna A, Stevenson K, Lawrence MS, Sougnez C, Stewart C, Sivachenko A, Wang L, (2013). Evolution and impact of subclonal mutations in chronic lymphocytic leukemia. Cell 152, 714–726. 10.1016/j.cell.2013.01.019. 23415222 PMC3575604

[43] Lawrence MS, Stojanov P, Mermel CH, Robinson JT, Garraway LA, Golub TR, Meyerson M, Gabriel SB, Lander ES, and Getz G (2014). Discovery and saturation analysis of cancer genes across 21 tumour types. Nature 505, 495–501. 10.1038/nature12912. 24390350 PMC4048962

[44] Carter SL, Cibulskis K, Helman E, McKenna A, Shen H, Zack T, Laird PW, Onofrio RC, Winckler W, Weir BA, (2012). Absolute quantification of somatic DNA alterations in human cancer. Nat. Biotechnol. 30, 413–421. 10.1038/nbt.2203. 22544022 PMC4383288

[45] McKenna A, Hanna M, Banks E, Sivachenko A, Cibulskis K, Kernytsky A, Garimella K, Altshuler D, Gabriel S, Daly M, and DePristo MA (2010). The Genome Analysis Toolkit: a MapReduce framework for analyzing next-generation DNA sequencing data. Genome Res. 20, 1297–1303. 10.1101/gr.107524.110. 20644199 PMC2928508

[46] Ramos AH, Lichtenstein L, Gupta M, Lawrence MS, Pugh TJ, Saksena G, Meyerson M, and Getz G (2015). Oncotator: cancer variant annotation tool. Hum. Mutat. 36, E2423–E2429. 10.1002/humu.22771. 25703262 PMC7350419

[47] Crowdis J, He MX, Reardon B, and Van Allen EM (2020). CoMut: visualizing integrated molecular information with comutation plots. Bioinformatics 36, 4348–4349. 10.1093/bioinformatics/btaa554. 32502231 PMC7520041

[48] Mermel CH, Schumacher SE, Hill B, Meyerson ML, Beroukhim R, and Getz G (2011). GISTIC2.0 facilitates sensitive and confident localization of the targets of focal somatic copy-number alteration in human cancers. Genome Biol. 12, R41. 10.1186/gb-2011-12-4-r41. 21527027 PMC3218867

[49] Rosenthal R, McGranahan N, Herrero J, Taylor BS, and Swanton C (2016). DeconstructSigs: delineating mutational processes in single tumors distinguishes DNA repair deficiencies and patterns of carcinoma evolution. Genome Biol. 17, 31. 10.1186/s13059-016-0893-4. 26899170 PMC4762164

[50] Leshchiner I, Mroz EA, Cha J, Rosebrock D, Spiro O, Bonilla-Velez J, Faquin WC, Lefranc-Torres A, Lin DT, Michaud WA, (2023). Inferring early genetic progression in cancers with unobtainable premalignant disease. Nat. Can. (Ott.) 4, 550–563. 10.1038/s43018-023-00533-. PMC10132986 37081260

[51] Robinson JT, Thorvaldsdóttir H, Winckler W, Guttman M, Lander ES, Getz G, and Mesirov JP (2011). Integrative genomics viewer. Nat. Biotechnol. 29, 24–26. 10.1038/nbt.1754. 21221095 PMC3346182

[52] Kircher M, Witten DM, Jain P, O’Roak BJ, Cooper GM, and Shendure J (2014). A general framework for estimating the relative pathogenicity of human genetic variants. Nat. Genet. 46, 310–315. 10.1038/ng.2892. 24487276 PMC3992975

[53] Adzhubei IA, Schmidt S, Peshkin L, Ramensky VE, Gerasimova A, Bork P, Kondrashov AS, and Sunyaev SR (2010). A method and server for predicting damaging missense mutations. Nat. Methods 7, 248–249. 10.1038/nmeth0410-248. 20354512 PMC2855889

[54] Fisher S, Barry A, Abreu J, Minie B, Nolan J, Delorey TM, Young G, Fennell TJ, Allen A, Ambrogio L, (2011). A scalable, fully automated process for construction of sequence-ready human exome targeted capture libraries. Genome Biol. 12, R1. 10.1186/gb-2011-12-1-r1. 21205303 PMC3091298

[55] Alexandrov LB, Kim J, Haradhvala NJ, Huang MN, Tian Ng AW, Wu Y, Boot A, Covington KR, Gordenin DA, Bergstrom EN, (2020). The repertoire of mutational signatures in human cancer. Nature 578, 94–101. 10.1038/s41586-020-1943-3. 32025018 PMC7054213

[56] Oliveira G, Egloff AM, Afeyan AB, Wolff JO, Zeng Z, Chernock RD, Zhou L, Messier C, Lizotte P, Pfaff KL, (2023). Preexisting tumor-resident T cells with cytotoxic potential associate with response to neoadjuvant anti-PD-1 in head and neck cancer. Sci. Immunol. 8, eadf4968. 10.1126/sciimmunol.adf4968. 37683037 PMC10794154

