# Supplementary material for: Genomic mediators of acquired resistance to immunotherapy in metastatic melanoma
Source: Cancer Cell. Author manuscript; Available in PMC 2025 Jul 7. (PMC12232206; doi:10.1016/j.ccell.2025.01.009)
Supplement: Genomic mediators of acquired resistance to immunotherapy in metastatic melanoma [file NIHMS2094197-supplement-Genomic_mediators_of_acquired_resistance_to_immunotherapy_in_metastatic_melanoma.pdf]

Cancer Cell, Volume *43*

Supplemental information

Genomic mediators of acquired resistance

to immunotherapy in metastatic melanoma

Julia Schiantarelli, Mouadh Benamar, Jihye Park, Haley E. Sax, Giacomo Oliveira, Alice Bosma-Moody, Katie M. Campbell, David Liu, Douglas B. Johnson, Scott Rodig, Catherine J. Wu, F. Stephen Hodi, Antoni Ribas, Eliezer Van Allen, and Rizwan Haq

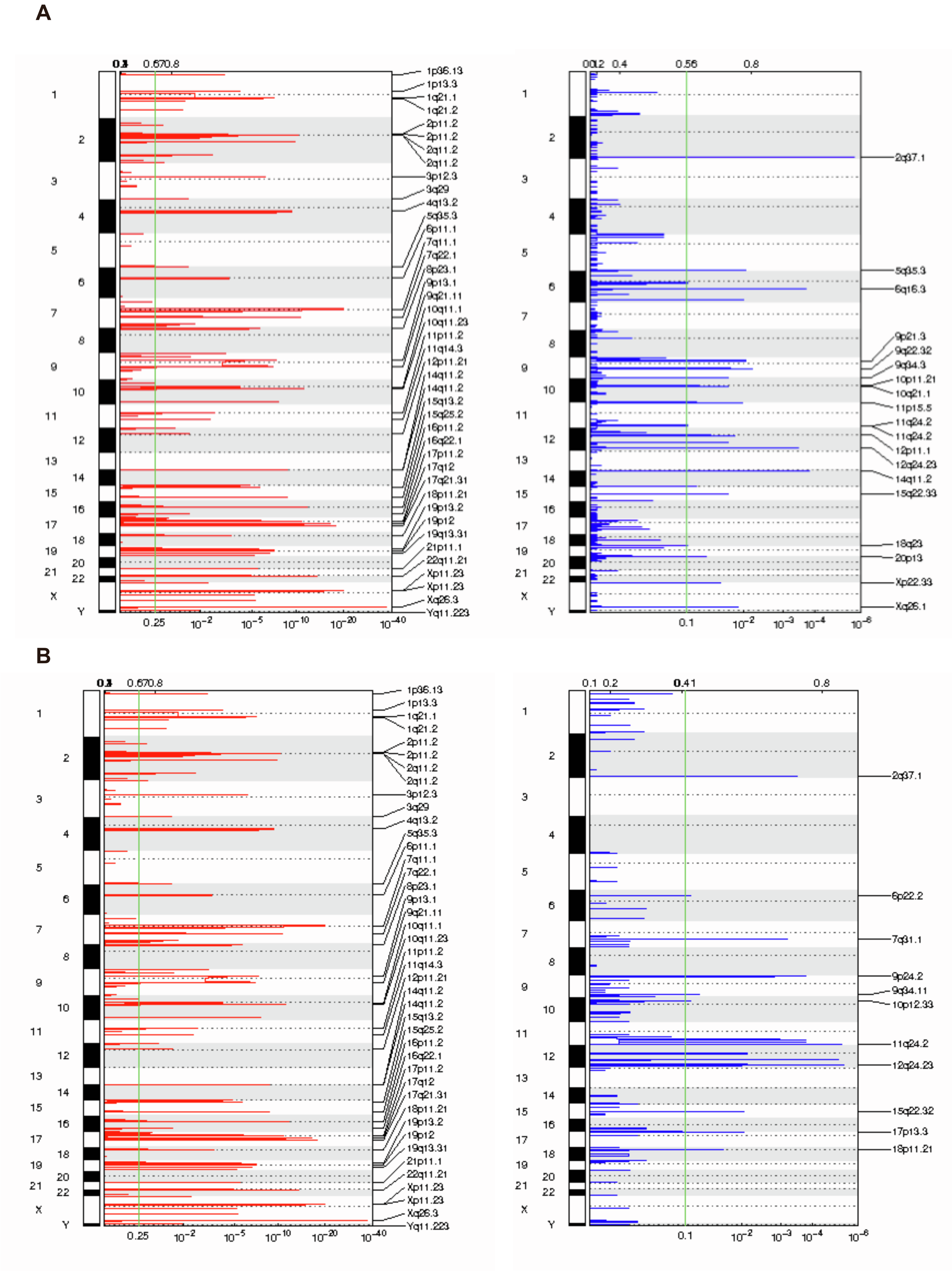
A
B
1p36.13
1p13.3
1q21.1
1q21.2
2p11.2
2q11.2
3p12.3
3q29
4q13.2
5q35.3
6p11.1
7q11.1
7q22.1
8p23.1
9p13.1
9q21.11
10q11.1
10q11.23
11p11.2
11q14.3
12p11.21
14q11.2
15q13.2
15q25.2
16p11.2
16q22.1
17p11.2
17q12
17q21.31
18p11.21
19p13.2
19p12
19q13.31
21p11.1
22q11.21
Xp11.23
Xq26.3
Yq11.223
2q37.1
5q35.3
6q16.3
9p21.3
9q22.32
9q34.3
10p11.21
10q21.1
11p15.5
11q24.2
12p11.1
12q24.23
14q11.2
15q22.33
18q23
20p13
Xp22.33
Xq26.1
6p22.2
7q31.1
9p24.2
9q34.11
10p12.33
15q22.32
17p13.3
18p11.21

C

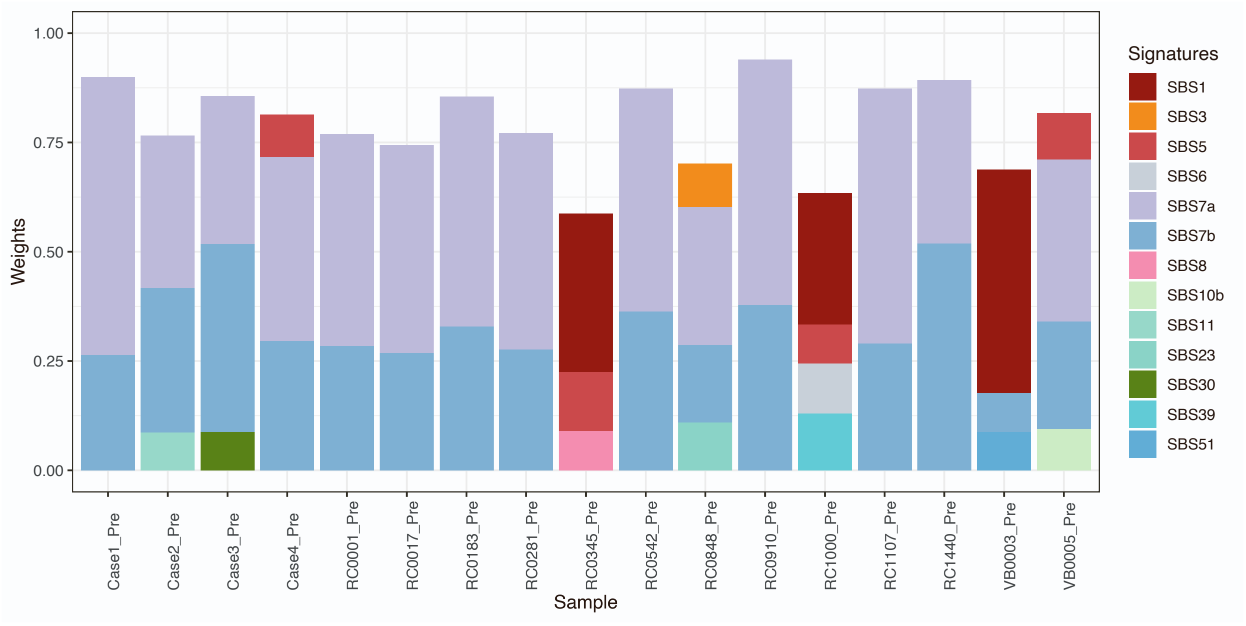

D

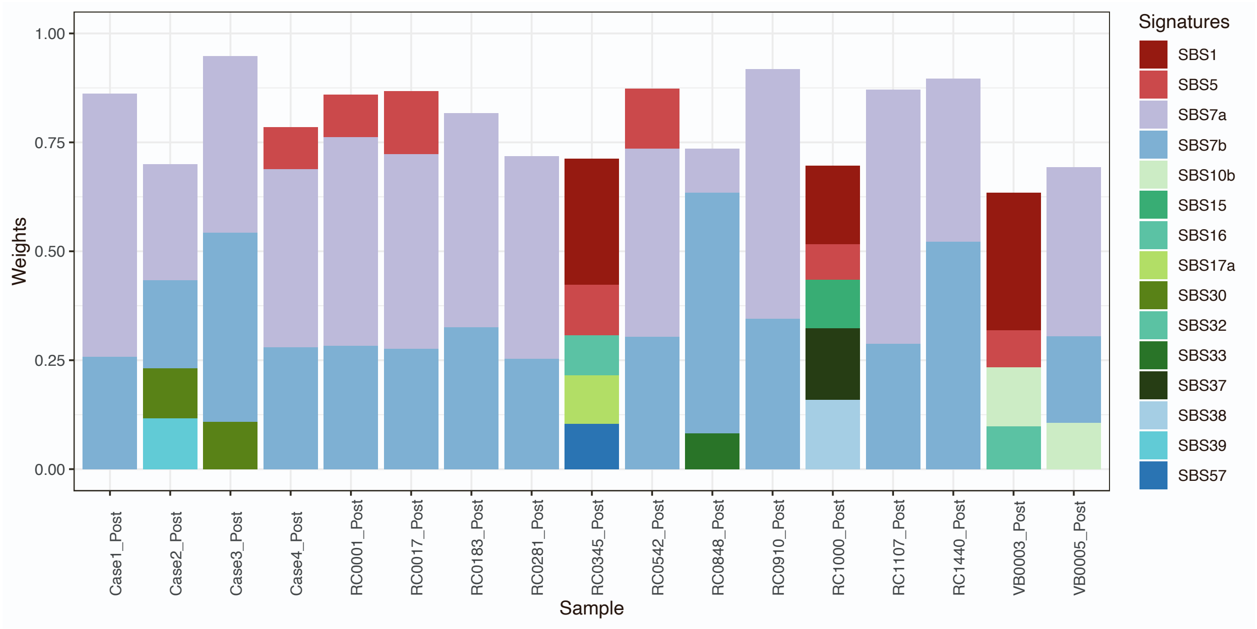

Figure S1. Comparison of copy number and mutational signature in pre- and post-treatment and resistant samples, related to Figure 2. (A-B) Arm-level and focal alterations in (A) pre-treatment or (B) post-treatment tumor recurrent amplifications (red) and deletions (blue) across the cohort identified using GISTIC2.0. (C-D) Cohort level mutational signatures in pre-treatment (C) and resistant samples (D) from COSMIC detected by deconstructSigs.

A

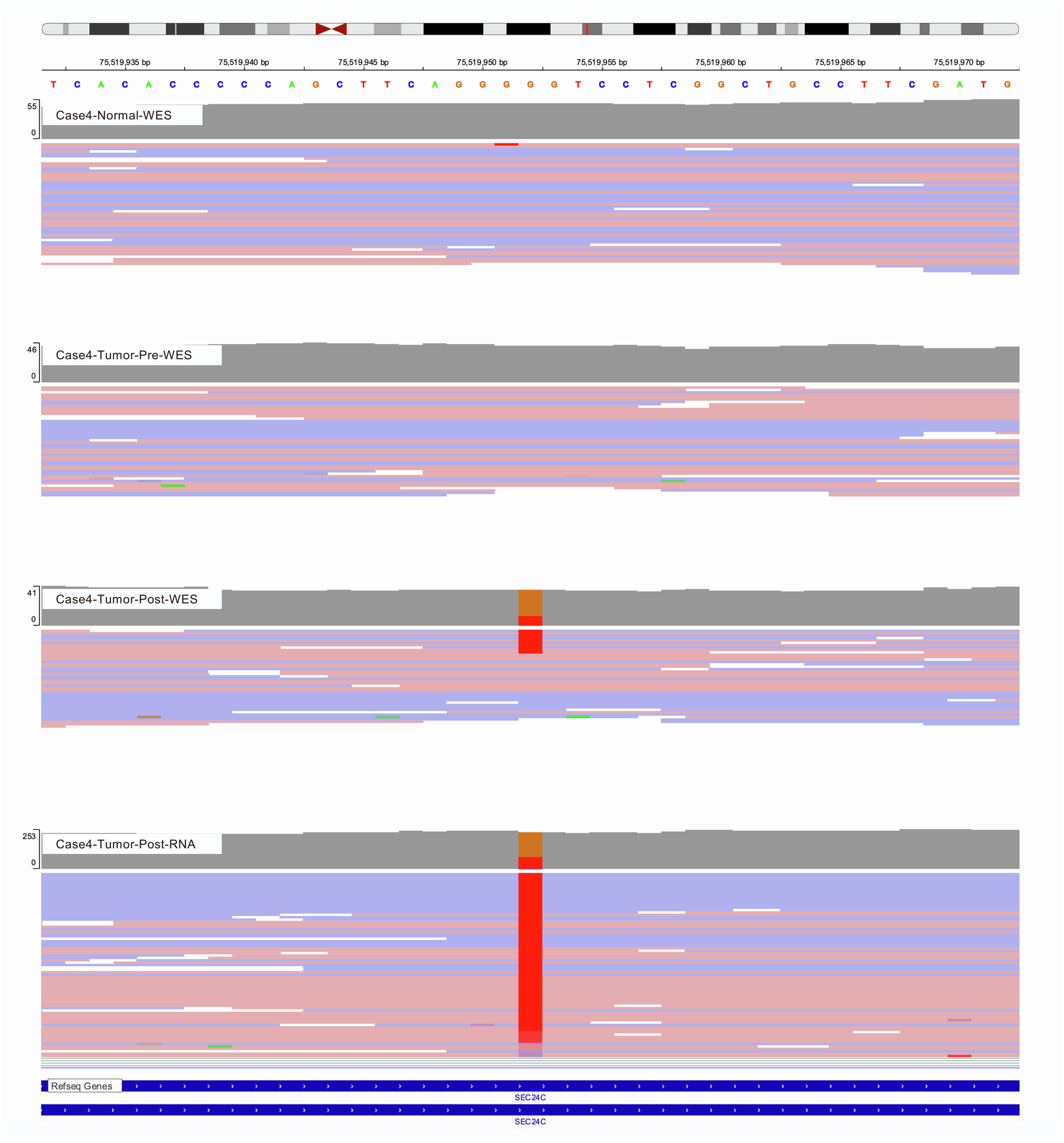
75,519,935 bp
75,519,940 bp
75,519,945 bp
75,519,950 bp
75,519,955 bp
75,519,960 bp
75,519,965 bp
75,519,970 bp
T C A C A C C C C C A G C T T C A G G G G G T C C T C G G C T G C C T T C G A T G
55
0
Case4-Normal-WES
46
0
Case4-Tumor-Pre-WES
41
0
Case4-Tumor-Post-WES
253
0
Case4-Tumor-Post-RNA
Refseq Genes
SEC24C
SEC24C

B

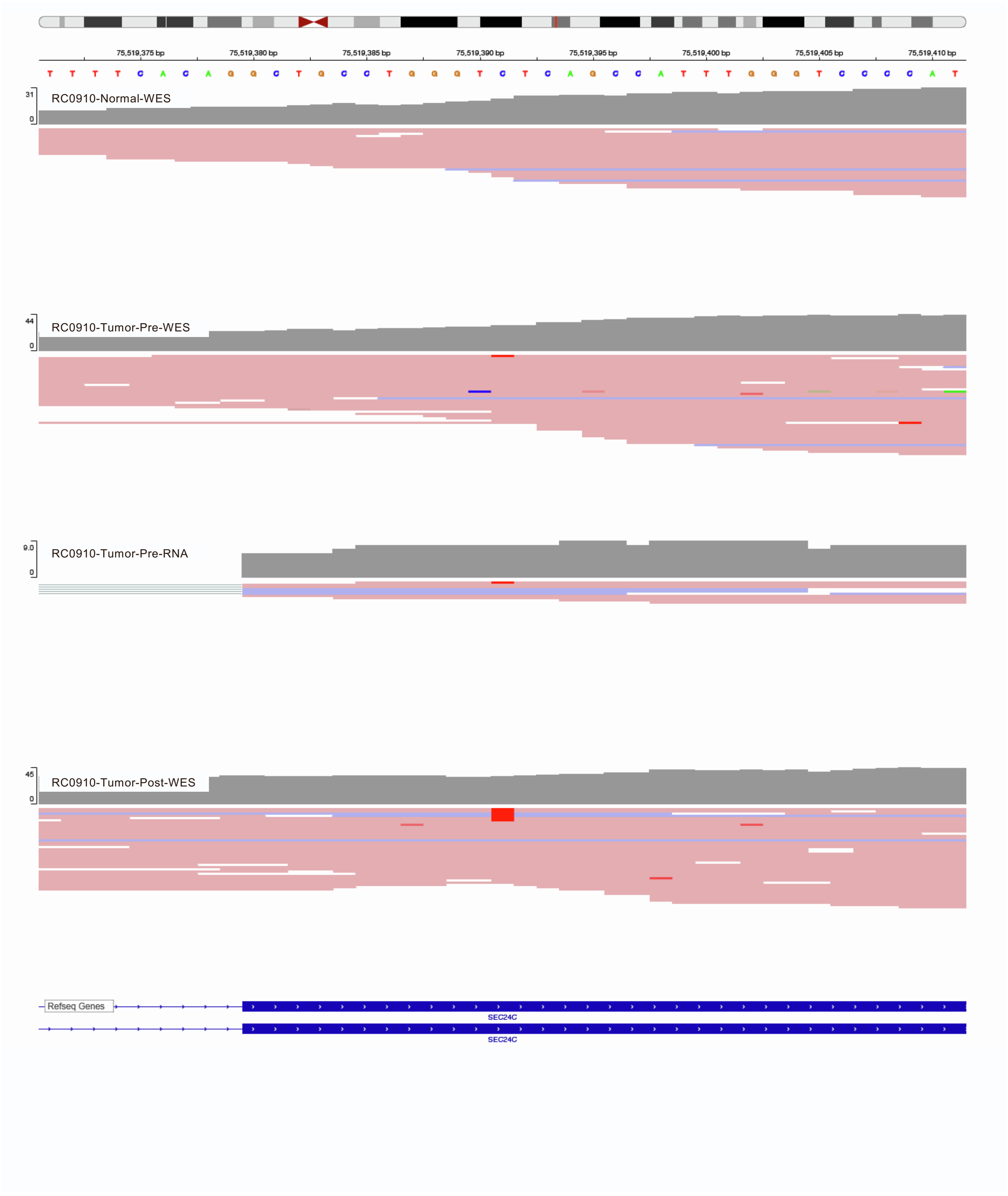

75,519,375 bp
75,519,380 bp
75,519,385 bp
75,519,390 bp
75,519,395 bp
75,519,400 bp
75,519,405 bp
75,519,410 bp
T T T T C A C A G G C T G C C T G G G T C T C A G C C A T T T G G G T C C C C A T
31
0
RC0910-Normal-WES
44
0
RC0910-Tumor-Pre-WES
9.0
0
RC0910-Tumor-Pre-RNA
45
0
RC0910-Tumor-Post-WES
Refseq Genes
SEC24C
SEC24C

C

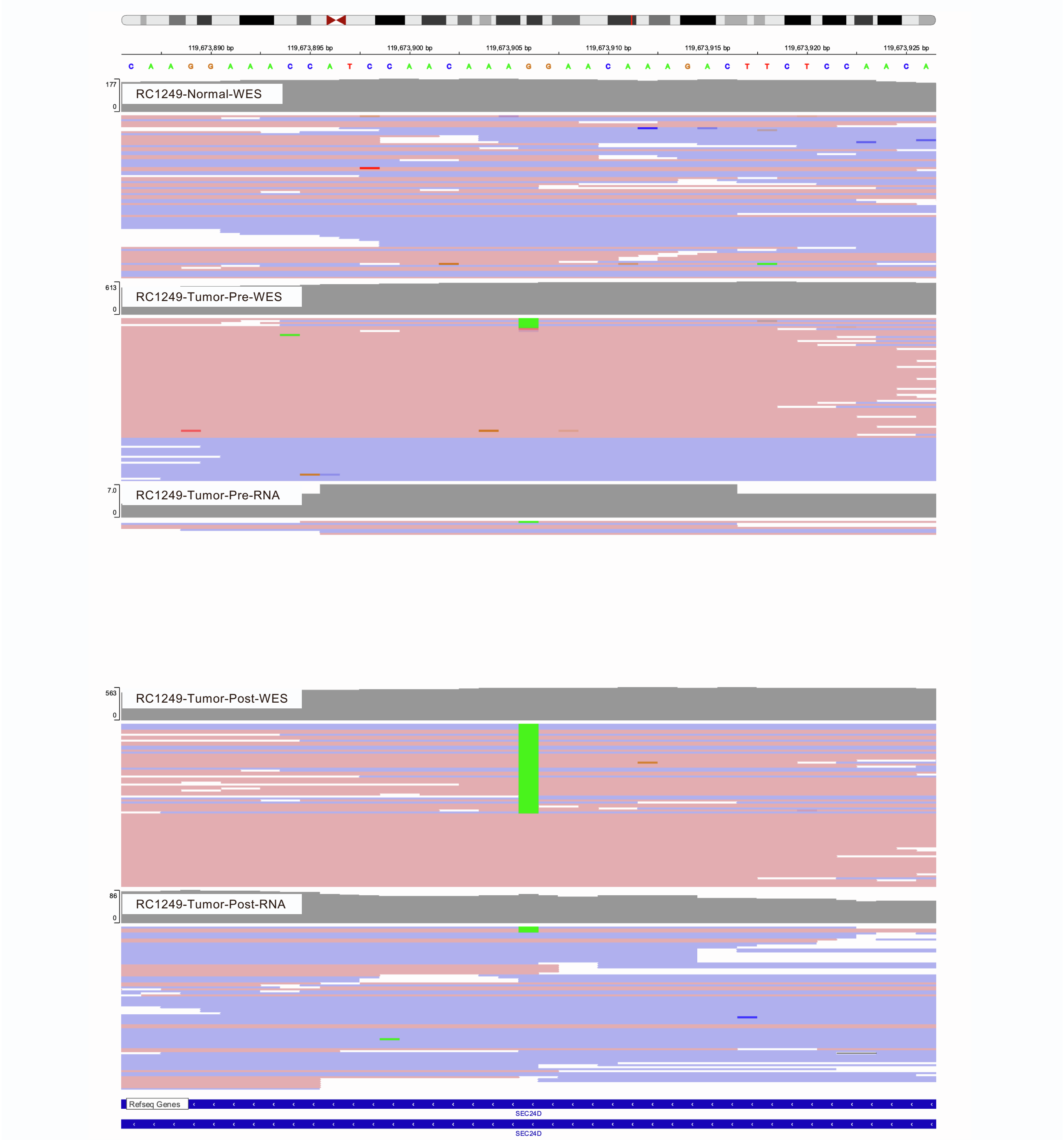

Figure S2. Integrative Genomics Viewer (IGV) snapshot highlighting *SEC24C* and *SEC24D* mutations, related to Figure 3. (A) IGV snapshot of *SEC24C* mutation (G>T, p.G220C, *SEC24C* chr 10 position 75519952) in Case 4 WES and RNA samples. Of note, the CCF of 0.08 was inferred by PhylogicNDT, but there were zero reads of the SEC24C mutation in the pre-treatment sample. (B) IGV snapshot of *SEC24C* mutation (C>T, p.S107F, *SEC24C* chr 10 position 75519391) in Case 0910 WES and RNA samples. RC0910 "Pre" samples had lower purity. (C) IGV snapshot of *SEC24D* mutation (G>A, p.P520L, *SEC24D* chr 4 position 119673906) in Case RC1249 WES and RNA samples. RC1249 "Pre" samples had lower purity than "Post" samples.

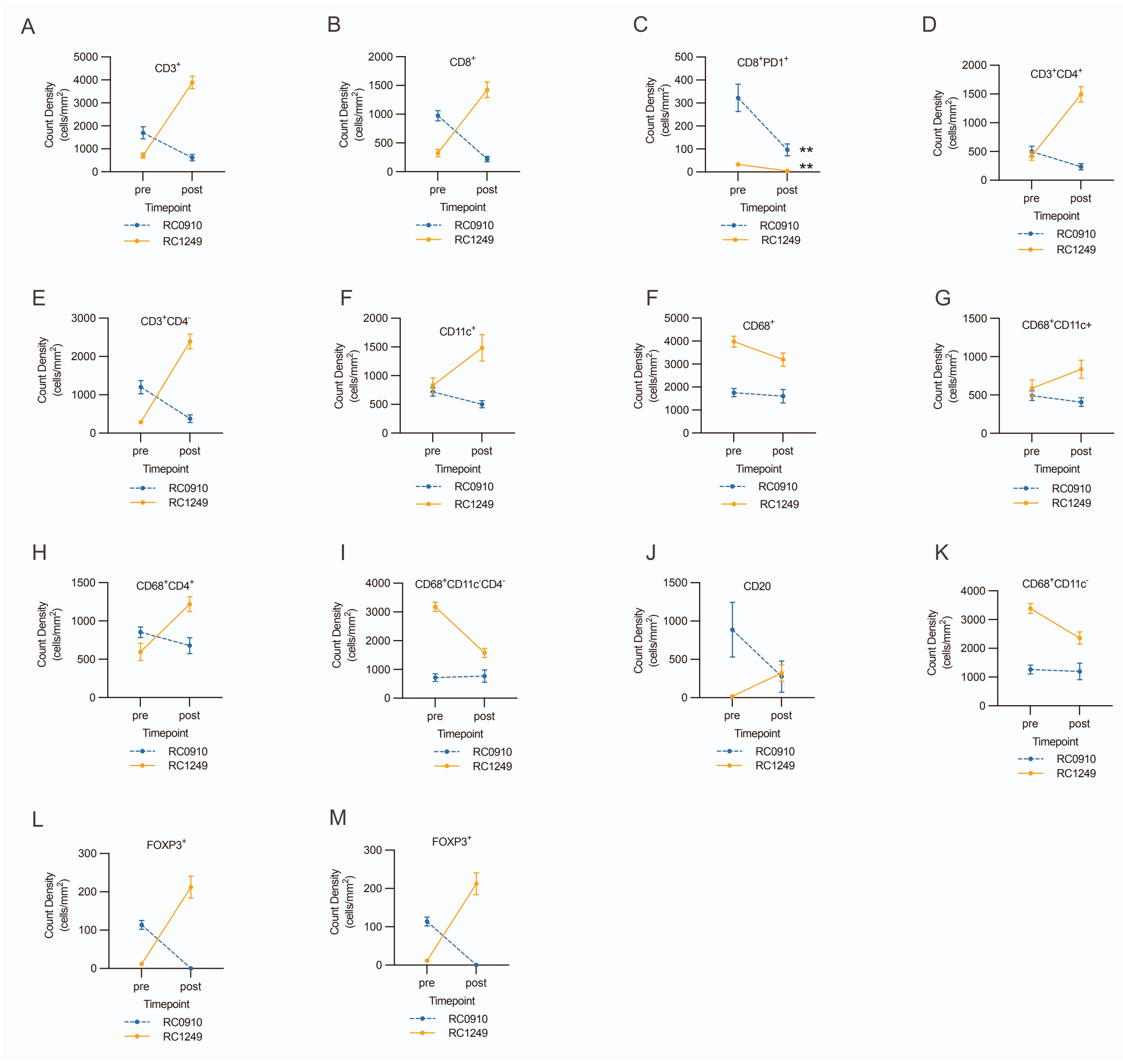

Figure S3. Quantification of immune cells in *SEC24* mutated tumors in pre-treatment and resistant biopsies, related to Figure 3. (A-M) Count density of indicated biomarkers using a supervised machine-learning algorithm (see Methods). The average cell densities (number of positive cells per mm^2) were calculated for each marker from 6 or 7 representative images per sample. Data are represented as the mean ± SEM across the images. Comparison of the mean between the “Pre” and “Post” samples was done using paired t-test. There were no shared trends between the mean differences between the two patients, except for the $CD8^+PD1^+$ cells (C). In both patients, $CD8^+PD1^+$ cells decreased in the “Post” sample compared to the “Pre” sample. **, P < 0.005.

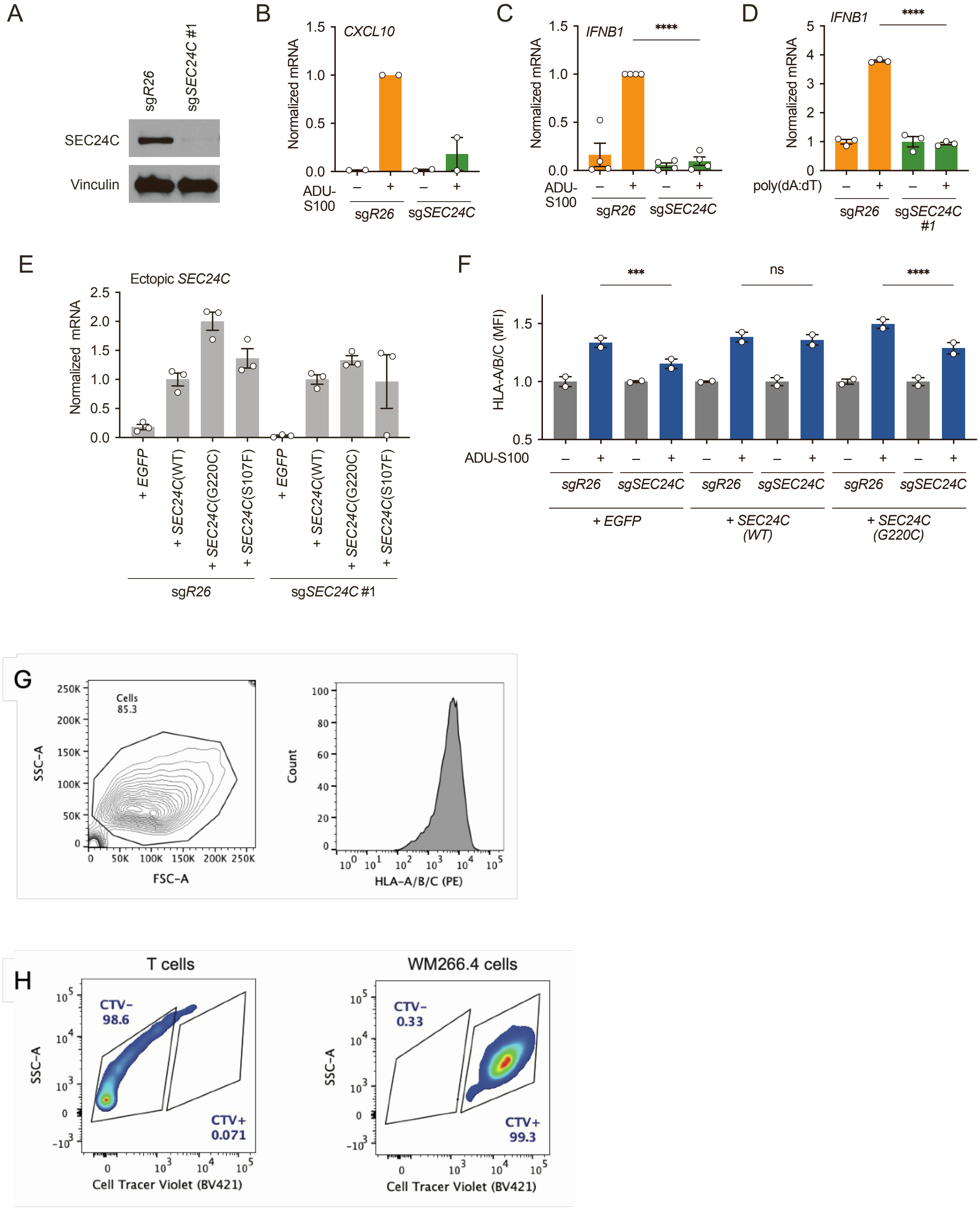

A
sgR26
sgSEC24C #1
SEC24C
Vinculin
B
CXCL10
Normalized mRNA
ADU-S100
sgR26
sgSEC24C
C
IFNB1
****
Normalized mRNA
ADU-S100
sgR26
sgSEC24C
D
IFNB1
****
Normalized mRNA
poly(dA:dT)
sgR26
sgSEC24C #1
E
Ectopic SEC24C
Normalized mRNA
+ EGFP
+ SEC24C(WT)
+ SEC24C(G220C)
+ SEC24C(S107F)
sgR26
sgSEC24C #1
F
***
ns
****
HLA-A/B/C (MFI)
ADU-S100
sgR26
sgSEC24C
+ EGFP
+ SEC24C (WT)
+ SEC24C (G220C)
G
Cells
85.3
SSC-A
FSC-A
Count
HLA-A/B/C (PE)
H
T cells
WM266.4 cells
CTV−
98.6
CTV+
0.071
CTV−
0.33
CTV+
99.3
SSC-A
Cell Tracer Violet (BV421)

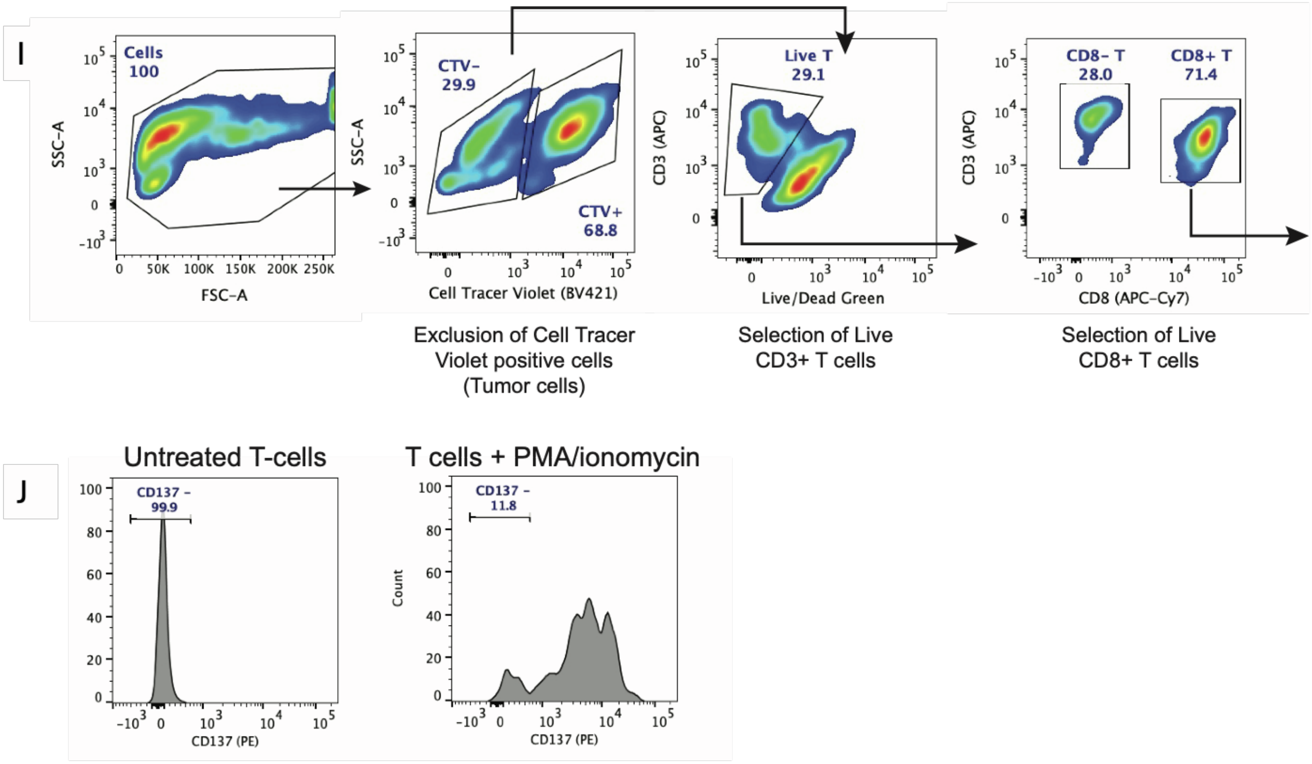

Figure S4. *SEC24C* mutations compromise interferon signaling, antigen presentation, and T cell activation, related to Figure 4. (A) Western blot showing knockout of *SEC24C* in THP-1 cells. (B-C) Relative *CXCL10* (B) and *IFNB1* (C) mRNA expression in control and *SEC24C*-deficient THP-1 cells following treatment with STING agonist ADU-S100 (20 µM) for 4 hours. (D) Relative *IFNB1* mRNA expression in control and *SEC24C*-deficient THP-1 cells following transfection with poly(dA:dT) (1 µg/ml). (E) Expression levels of ectopically expressed *SEC24C* wild-type and mutants in WM266.4 cells. (F) Relative HLA expression in WM266.4 cells expressing *EGFP*, wild-type, or mutant *SEC24C* in control and *SEC24C*-deficient cells following treatment with ADU-S100 for 24h. (G) Gating strategy used to analyze HLA (A/B/C) presentation in WM266.4 melanoma cells. (H-J) T cell killing assay controls and gating strategy. (H) Melanoma cells after 20 min labeling with Cell Trace Violet. T cells are not stained and serve as a negative control. (I) Gating strategy to analyze MLANA-specific $CD8^+$ or $CD4^+$ ($CD8^-$) T cell activation after co-culture with WM266.4 melanoma cells. (J) $CD137^+$ $CD8^+$ T cells with or without PMA/ionomycin. For all panels, two-way ANOVA with Sidak multiple comparisons test was used to determine statistical significance. ****, P <0.0001; ***, P < 0.0005; **, P < 0.005; *, P < 0.05; ns, no significance.
